# A multiview video–text model detects obstructive coronary stenosis and predicts 1-year cardiovascular events from angiography

**DOI:** 10.1038/s44161-026-00890-8

**Published:** 2026-10-13

**Authors:** Sarra Harrabi, Yichen Wu, Omar Majwal, Gabriel Asslo, George Azar, Geoffrey H. Tison, Minhaj Ansari, Milos Vukadinovic, David Ouyang, Joshua P. Barrios, Jacques Delfrate, Robert Avram

**Affiliations:** 1 Division of Cardiology, Department of Medicine, Montreal Heart Institute, Université de Montréal, Montreal, Quebec Canada; 2 HeartWise.ai, Montreal, Quebec Canada; 3 Faculty of Engineering, McGill University, Montreal, Quebec Canada; 4 Department of Medicine, Université de Montréal, Montreal, Quebec Canada; 5 Division of Cardiology, Department of Medicine, University of California, San Francisco, CA USA; 6 Cardiovascular Research Institute, University of California, San Francisco, CA USA; 7 Bakar Computational Health Sciences Institute, University of California, San Francisco, CA USA; 8 Department of Cardiology, Smidt Heart Institute, Cedars-Sinai Medical Center, Los Angeles, CA USA; 9 Department of Bioengineering, University of California Los Angeles, Los Angeles, CA USA; 10 Department of Medicine, Kaiser Permanente, San Francisco, CA USA

**Keywords:** Interventional cardiology, Ischaemia

## Abstract

Invasive coronary angiography is the reference standard for diagnosing coronary artery disease, yet assessment is subjective, and existing artificial-intelligence tools typically assess stenosis in individual frames or videos and do not integrate the full multiview angiographic study. Here we show that DeepCORO-CLIP, trained on 203,808 angiographic videos and reports from 28,117 patients, interprets whole multiple-video angiograms at once. It detects obstructive stenoses (≥70%), with an area under the receiver operating characteristic curve (AUROC) of 0.89 internally and externally, and identifies chronic total occlusion (AUROC = 0.90 to 0.95), intra-coronary thrombus and calcification (AUROC = 0.90 to 0.91). It also predicts 1-year cardiovascular events (AUROC = 0.79, 95% CI of 0.74 to 0.84, versus 0.65 for a model derived using clinical variables) and left ventricular ejection fraction (mean absolute error 7.3% versus 8.8% for a previous model), both achieved using coronary angiography alone. In a blinded review of 365 evaluations across 288 angiograms, its reports were clinically usable in 76% of evaluations versus 88% for cardiologists, with comparable false positives (17% versus 14%, *P* = 0.38); residual missed stenoses reflected an adjustable threshold rather than an absent signal.

## Abstract (web summary)

Harrabi et al. develop DeepCORO-CLIP, a multiview video–text model, to interpret coronary angiograms by detecting obstructive stenosis and predict 1-year cardiovascular events.

## Main

Coronary artery disease (CAD) remains the leading cause of morbidity and mortality worldwide, with invasive coronary angiography (ICA) serving as the reference standard for diagnostic evaluation and therapeutic decision-making^1^. Despite its central role in cardiovascular medicine, visual interpretation of coronary angiography has notable limitations that impact clinical outcomes. Visual stenosis assessment on ICA shows substantial intra- and interobserver variability ranging from 7.6% to 22.5%, with even greater variability in single-physician settings, leading to overestimation of stenosis severity in over a quarter of cases and potentially contributing to inappropriate coronary interventions in 17% of bypass surgery cases and 10% of stent procedures^2–4^. These limitations underscore the urgent need for objective, reproducible methods to enhance coronary angiography interpretation and improve patient care.

Artificial intelligence (AI) has appeared as a promising solution to address the inherent limitations of visual coronary angiography assessment. Early single-view approaches showed substantial potential, with systems like CathAI achieving an area under the receiver operating characteristic curve (AUROC) of 0.86 for epicardial stenoses detection through sequential neural networks for projection angle classification, anatomic structure identification and stenosis estimation^5^. Subsequent advances moved toward video-based analysis, exemplified by DeepCORO, a video-based deep learning model that achieved a mean absolute error (MAE) of 20.2% in stenosis percentage prediction, showed lower variability than clinical reports (19.1% versus 21.0%) and highlighted the contribution of temporal information to angiographic analysis^6^.

Despite these advances, current AI approaches for coronary angiography face critical limitations. Existing models operate on single videos or frames, process individual stenosis lesions through multistep pipelines rather than a single model trained end-to-end, and are trained with purely supervised objectives for a single task, typically stenosis estimation^5,6^. This task-specific, single-view paradigm limits both generalizability and the quality of learned representations, precluding comprehensive coronary evaluation across multiple interpretation end points such as chronic total occlusion (CTO) detection, thrombus identification, calcification assessment and prognostic evaluation^6^. Visual language models are reshaping medical imaging AI by learning a shared representation between pixels and clinical language, typically via contrastive objectives that scale across institutions, scanners and reporting styles^7,8^. This paradigm moves the field beyond single-task classifiers toward foundation models that can support zero-shot transfer, multitask adaptation, retrieval and report-conditioned reasoning. In echocardiography, EchoCLIP^8^ showed large-scale video text alignment for robust, externally validated interpretation, and EchoPrime extended the approach with explicit multivideo, multiview fusion to better reflect study-level clinical reasoning^7^.

To address these limitations, we present DeepCORO-CLIP, a multiview model trained with video–text contrastive learning for comprehensive coronary angiography interpretation. We evaluated whether DeepCORO-CLIP improves performance across core clinical tasks including stenosis quantification, CTO detection, thrombus identification and calcification grading. We further assessed the model’s capacity for tasks beyond standard angiographic interpretation, including left ventricular ejection fraction (LVEF) estimation and 1-year major adverse cardiovascular event (MACE) prediction from angiographic videos alone. We also evaluated whether the learned study-level embeddings encode clinically meaningful representations of coronary anatomy capable of detecting interventional changes and tracking longitudinal disease progression across serial examinations. Finally, in a prospective, blinded reader study, we evaluated the standalone clinical quality of the model’s automatically generated free-text reports against expert operator reports.

## Results

**Fig. 1:**
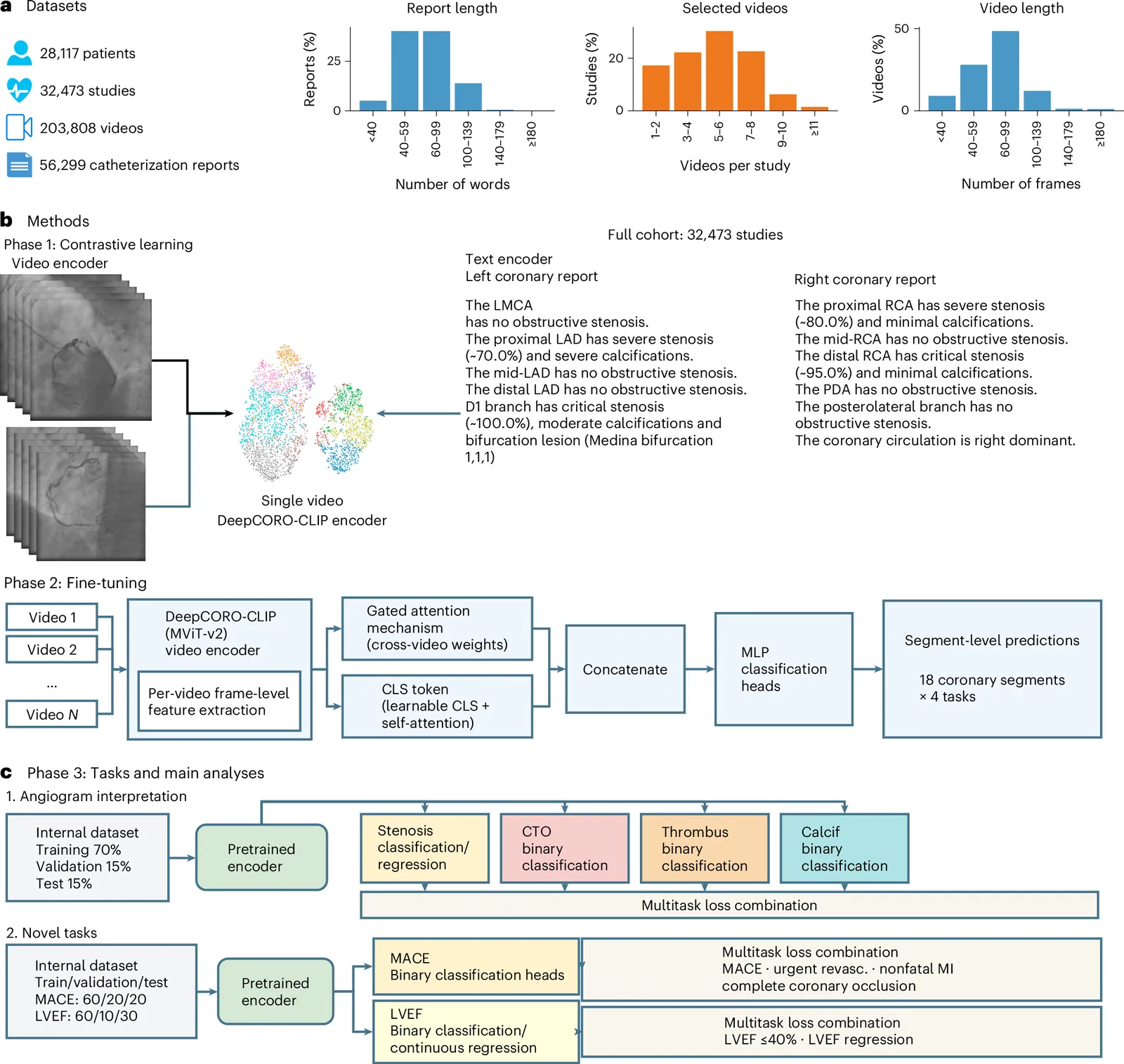
DeepCORO-CLIP: a vision-language model for coronary angiography interpretation. **a**, Dataset characteristics. The internal cohort comprised 28,117 patients with 32,473 studies, yielding 203,808 angiography videos paired with 56,299 catheterization reports. The report-length histogram represents 62,805 distinct study/report–text pairs, deduplicated within study. The selected-videos-per-study histogram represents 167,134 selected diagnostic videos across all 32,473 studies. These derived units differ from the broader source-cohort counts of 56,299 reports and 203,808 videos. The frame-count histogram includes all 167,134 selected videos, with no missing frame counts. The mean number of selected videos per study is 5.15. Histogram bars show percentages per bin, including an open-ended final bin; heights are proportions rather than probability densities. No error bars or hypothesis tests are shown. **b**, Model architecture and training phases. Phase 1 uses contrastive learning to align video embeddings from the Multiscale Vision Transformer version 2 (MViT-v2) encoder with corresponding text descriptions from catheterization reports. Phase 2 fine-tunes the pretrained encoder using a multi-instance learning framework with gated attention to aggregate frame-level features across multiple videos, generating segment-level predictions for 18 coronary segments across 4 tasks. **c**, Downstream tasks and analyses. Task 1 evaluates angiogram interpretation including stenosis severity, CTO detection, thrombus identification and calcification grading using multitask learning (70/15/15 train/validation/test split). Task 2 assesses novel predictive capabilities for MACE (urgent revascularization, nonfatal MI, complete coronary occlusion) and LVEF estimation using independent train/validation/test splits (60/20/20 for MACE and 60/10/30 for LVEF). D1, diagonal branch 1; CLS, classification token; MLP, multilayer perceptron; Calcif, calcification; revasc., revascularization; LMCA, left main coronary artery. Medina bifurcation 1,1,1 denotes a coronary bifurcation lesion involving the proximal main vessel, distal main vessel and side branch, with 1 indicating disease in each segment. Source data

**Extended Data Fig. 1:**
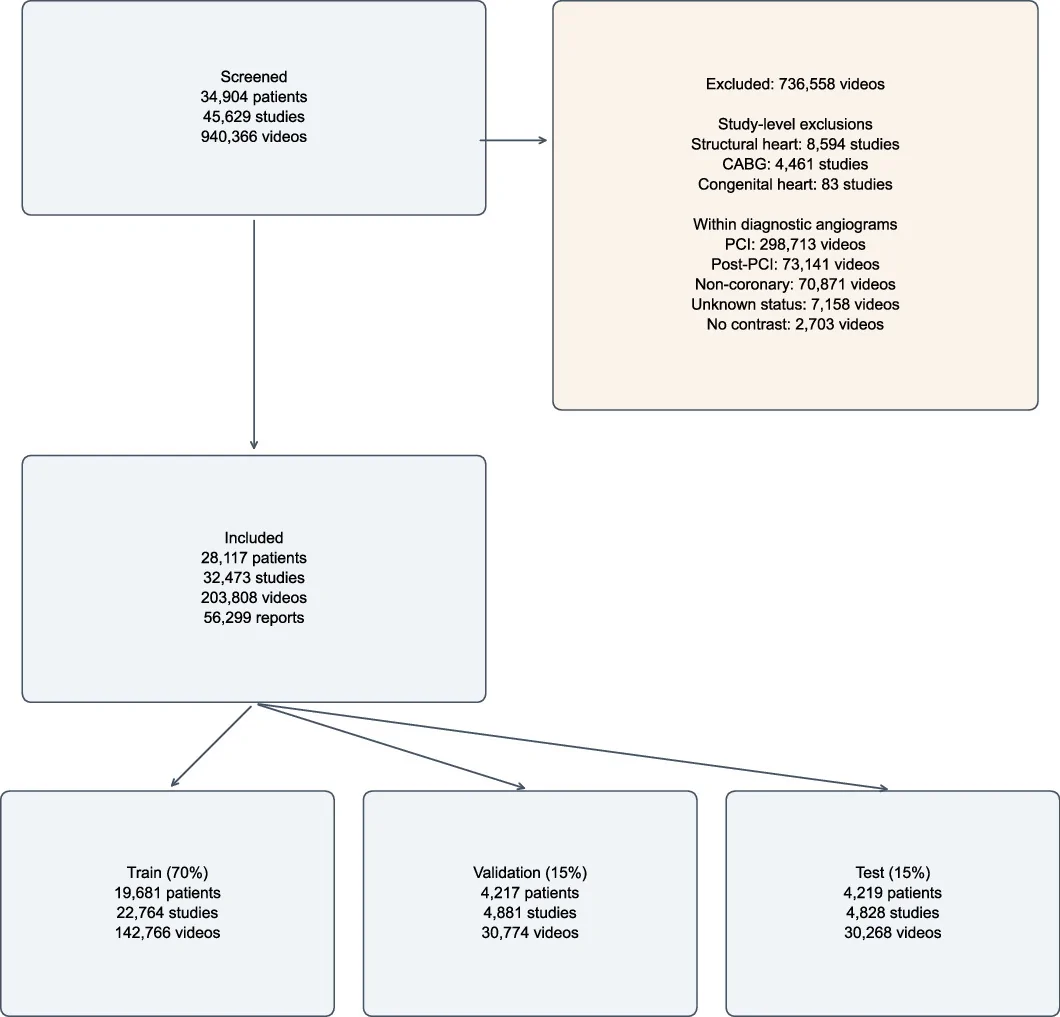
Cohort selection and study allocation. Patient-level training, validation and test split: 70%, 15% and 15%, respectively. Overall video exclusion counts include study-level exclusions. The listed video-level exclusion categories apply within diagnostic angiograms and do not constitute the entire exclusion total. Source data

Of 940,366 video–report pairs screened, 736,558 (78.3%) were excluded because of procedural equipment present, absent contrast, previous coronary artery bypass grafting (CABG) or acquisition after percutaneous coronary intervention (PCI). The final dataset comprised 203,808 videos from 28,117 patients, covering 32,473 studies and 56,299 reports spanning the left coronary artery (LCA) and right coronary artery (RCA), split into training (70%), validation (15%) and test (15%) sets with patient-level stratification (Fig. 1a and Extended Data Fig. 1). Patient demographics and angiographic characteristics are presented in Table 1. Mean age was 67.3 years, with standard deviation (s.d.) of 11.6 and with 66.6% male patients. Comorbidities included hypertension (69.6%), diabetes (27.1%), chronic kidney disease (8.4%), atrial fibrillation (13.4%) and current or previous smoking (21.8%), and previous PCI was documented in 35.1% of patients. Baseline characteristics were well balanced across training, validation and test sets, with no statistically significant differences (all *P* > 0.05) and a maximum absolute difference of 1.5 percentage points (current or previous smoking). Studies averaged 4.7 videos (s.d. 1.4) acquired at different angles, 493.1 frames per study (s.d. 239.4) and a frame rate of 11.7 frames per s (s.d. 3.6).

**Table 1:** Baseline patient and study characteristics

Characteristic	Training	Validation	Test	*P* value
Number of studies	22,764	4,881	4,828	NA
Number of patients	19,681	4,217	4,219	NA
Number of videos	142,766	30,774	30,268	NA
Demographics				
Age, years, mean ± s.d.	67.2 ± 11.7 (*n* = 19,160)	67.2 ± 11.9 (*n* = 4,092)	67.3 ± 11.6 (*n* = 4,085)	0.777
Men, *n* (%)	12,784 (66.5%)	2,702 (65.8%)	2,731 (66.6%)	0.656
Women, *n* (%)	6,446 (33.5%)	1,406 (34.2%)	1,370 (33.4%)	0.656
Missing, *n* (%)	451 (2.3%)	109 (2.6%)	118 (2.8%)	
Medical history (patient level)				
Hypertension, *n* (%)	11,477 (70.5%)	2,432 (70.1%)	2,417 (69.6%)	0.516
Diabetes, *n* (%)	4,485 (28.0%)	939 (27.6%)	924 (27.1%)	0.494
Chronic kidney disease, *n* (%)	1,358 (9.0%)	278 (8.6%)	268 (8.4%)	0.411
Atrial fibrillation, *n* (%)	2,142 (13.5%)	462 (13.5%)	452 (13.4%)	0.977
Current or previous smoking, *n* (%)	3,462 (21.8%)	685 (20.3%)	738 (21.8%)	0.135
Previous PCI (prior stent), *n* (%)	6,831 (34.9%)	1,439 (34.2%)	1,473 (35.1%)	0.663
Imaging system vendor (study level)				
Philips, *n* (%)	10,846 (47.7%)	2,350 (48.2%)	2,251 (46.7%)	0.686
Siemens, *n* (%)	7,321 (32.2%)	1,549 (31.7%)	1,609 (33.4%)	
Toshiba, *n* (%)	4,578 (20.1%)	980 (20.1%)	964 (20.0%)	
Zonare, *n* (%)	1 (0.0%)	0 (0.0%)	0 (0.0%)	
Vendor unresolved, *n*	16	2	4	
Video characteristics				
Frames per s, mean ± s.d.	11.7 ± 3.6 (*n* = 8,381)	11.6 ± 3.5 (*n* = 1,785)	11.7 ± 3.6 (*n* = 1,851)	0.837
Frames per study, mean ± s.d.	492.8 ± 242.7 (*n* = 2,656)	508.6 ± 244.8 (*n* = 568)	493.1 ± 239.4 (*n* = 589)	0.363
Views per study, mean ± s.d.	4.8 ± 1.4	4.8 ± 1.4	4.7 ± 1.4	0.487

Percentages are computed over patients (or studies, for study-level rows) with nonmissing data for the listed variable; *n* with missing data is reported where applicable. *P* values compare the training, validation and test sets, using the two-sample *t*-test (or Wilcoxon rank-sum test when normality was not satisfied) for continuous variables and the chi-square test (or Fisher exact test when expected cell counts were small) for categorical variables; all tests are two-sided at an alpha of 0.05 without adjustment for multiple comparisons.

NA, not applicable.

### Model training performance

**Extended Data Fig. 2:**
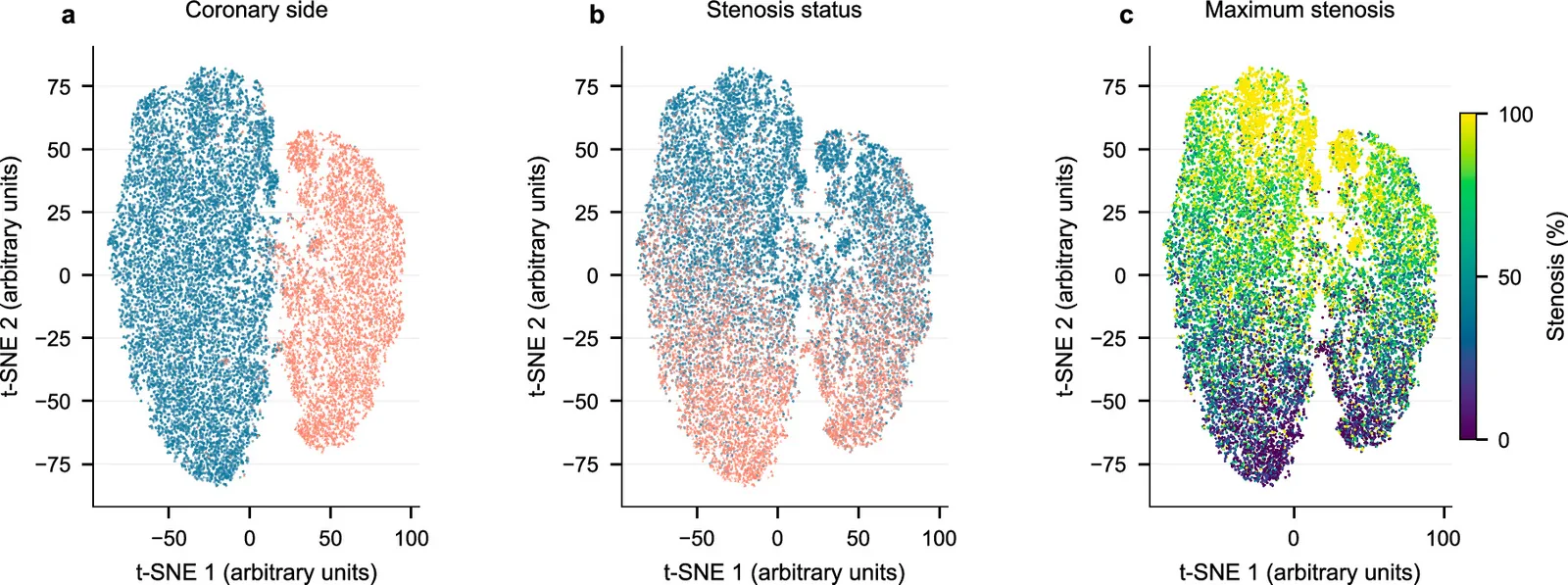
Video embedding visualization. Video-encoder embeddings. n = 24,708 videos; each point represents one video. **a**, Blue circles, left coronary videos; coral triangles, right coronary videos. **b**, Blue circles, abnormal stenosis ( ≥ 50%); coral triangles, normal. **c**, Colour encodes maximum stenosis (%); the 0 to 100 scale represents percentage stenosis. Embeddings are visualized using t-SNE; axes have arbitrary units. No hypothesis test was applied. Source data

Pretraining of the DeepCORO-CLIP video encoder ran for 30 epochs (28.8 h on a single graphics processing unit (GPU), with peak memory of 90.7 GB). We used cross-modal retrieval as a prompt-free, zero-shot probe of contrastive alignment: on 24,708 held-out videos and 4,851 unique reports, video-to-text Recall@1/5/10 was 0.07/0.13/0.17 (median rank 156), and text-to-video Recall@1/5/10 was 0.01/0.04/0.07, both more than two orders of magnitude above chance. As further prompt-free evidence, study-level cosine similarity discriminated same-patient videos from different-patient angiogram exam pairs with AUROC of 0.76 (median cosine 0.52 versus 0.13), despite no training on patient identity. The learned 512-dimensional embedding space showed clinically interpretable, unsupervised organization: near-complete left versus right coronary separation (93% to 99% cluster purity) and a clear severity gradient from normal (peak stenosis 12% to 18%) to severe disease (72% to 82%), and *k*-means clustering (*k* = 12) recovered anatomically and pathologically coherent subgroups spanning normal, mild, moderate and severe disease in both coronary systems (Supplementary Table 6 and Extended Data Fig. 2).

### Primary outcome results

DeepCORO-CLIP quantified continuous stenosis with MAE of 9.0% (LCA) and 9.9% (RCA) and Pearson *r* of 0.49 and 0.59 (Table 2). Accuracy was segment dependent, with stronger correlations in proximal segments (proximal RCA *r* = 0.73, left anterior descending artery (LAD) *r* = 0.64, left circumflex artery (LCX) *r* = 0.56) than in distal or low-prevalence segments (distal LAD *r* = 0.01, distal LCX *r* = 0.12, posterior descending artery (PDA) *r* = 0.21), tracking both disease prevalence (proximal LAD 16.8% and RCA 16.3% versus distal LAD 3.3% and LCX 1.9%) and small-caliber visualization limits (Extended Data Table 1). For obstructive stenosis (≥70%), micro-AUROC was 0.89 with a 95% confidence interval (CI) of 0.88 to 0.89 (sensitivity 0.85, specificity 0.70 at the Youden index; Table 2). The model also detected moderate-to-severe calcification (AUROC 0.90 LCA, 0.91 RCA; Extended Data Table 2), thrombus despite 0.25% prevalence (AUROC 0.90, 95% CI 0.87 to 0.92; sensitivity 0.74, specificity 0.92; Extended Data Table 2) and CTO (AUROC 0.90 LCA, 0.95 RCA; sensitivity 0.76 and 0.91; Extended Data Table 2).

**Table 2:** Consolidated performance metrics by coronary segment with 95% CIs

Task	Metric	Global (All)	LCA	RCA
Stenosis (regression)	*n* (abnormal/total)	20,844/86,544 (24.1%)	13,834/62,504 (22.1%)	7,010/24,040 (29.2%)
	MAE (95% CI)	9.2 (9.1–9.4)	9.0 (8.8–9.1)	9.9 (9.6–10.1)
	Pearson *r* (95% CI)	0.53 (0.52–0.53)	0.49 (0.48–0.50)	0.59 (0.58–0.61)
Stenosis ≥ 70%	*n* (abnormal/total)	7,270/86,544 (8.4%)	5,024/62,504 (8.0%)	2,246/24,040 (9.3%)
	AUROC (95% CI)	0.89 (0.88–0.89)	0.88 (0.88–0.89)	0.91 (0.90–0.91)
	AUPRC (95% CI)	0.52 (0.50–0.53)	0.48 (0.47–0.50)	0.59 (0.57–0.61)
	Sens (95% CI)	0.85 (0.84–0.86)	0.83 (0.82–0.84)	0.89 (0.87–0.90)
	Spec (95% CI)	0.70 (0.70–0.71)	0.70 (0.70–0.70)	0.71 (0.70–0.71)
Calcification	*n* (abnormal/total)	1,685/86,544 (1.9%)	1,141/62,504 (1.8%)	544/24,040 (2.3%)
	AUROC (95% CI)	0.90 (0.90–0.91)	0.90 (0.89–0.91)	0.91 (0.90–0.92)
	AUPRC (95% CI)	0.18 (0.17–0.20)	0.18 (0.17–0.21)	0.18 (0.16–0.21)
	Sens (95% CI)	0.85 (0.83–0.87)	0.83 (0.81–0.85)	0.89 (0.86–0.91)
	Spec (95% CI)	0.80 (0.80–0.81)	0.81 (0.81–0.81)	0.79 (0.78–0.79)
CTO	*n* (abnormal/total)	550/86,544 (0.6%)	273/62,504 (0.4%)	277/24,040 (1.2%)
	AUROC (95% CI)	0.92 (0.91–0.93)	0.90 (0.88–0.92)	0.95 (0.93–0.96)
	AUPRC (95% CI)	0.18 (0.15–0.21)	0.10 (0.07–0.13)	0.27 (0.22–0.32)
	Sens (95% CI)	0.84 (0.81–0.87)	0.76 (0.71–0.81)	0.91 (0.88–0.94)
	Spec (95% CI)	0.86 (0.86–0.86)	0.87 (0.87–0.87)	0.84 (0.83–0.84)
Thrombus	*n* (abnormal/total)	217/86,544 (0.3%)	113/62,504 (0.2%)	104/24,040 (0.4%)
	AUROC (95% CI)	0.90 (0.87–0.92)	0.88 (0.85–0.91)	0.92 (0.89–0.95)
	AUPRC (95% CI)	0.23 (0.17–0.29)	0.17 (0.10–0.26)	0.30 (0.21–0.40)
	Sens (95% CI)	0.74 (0.68–0.80)	0.61 (0.52–0.70)	0.88 (0.81–0.93)
	Spec (95% CI)	0.92 (0.92–0.92)	0.95 (0.95–0.95)	0.83 (0.83–0.84)

Global (all) metrics represent sample-weighted averages across LCA and RCA territories. Values are point estimates in the internal held-out test set (*n* = 4,828 studies) with 95% CIs from 1,000 bootstrap iterations; sensitivity and specificity are reported at the Youden index; no hypothesis test is reported in this table.

Sens, sensitivity; Spec, specificity.

**Fig. 2:**
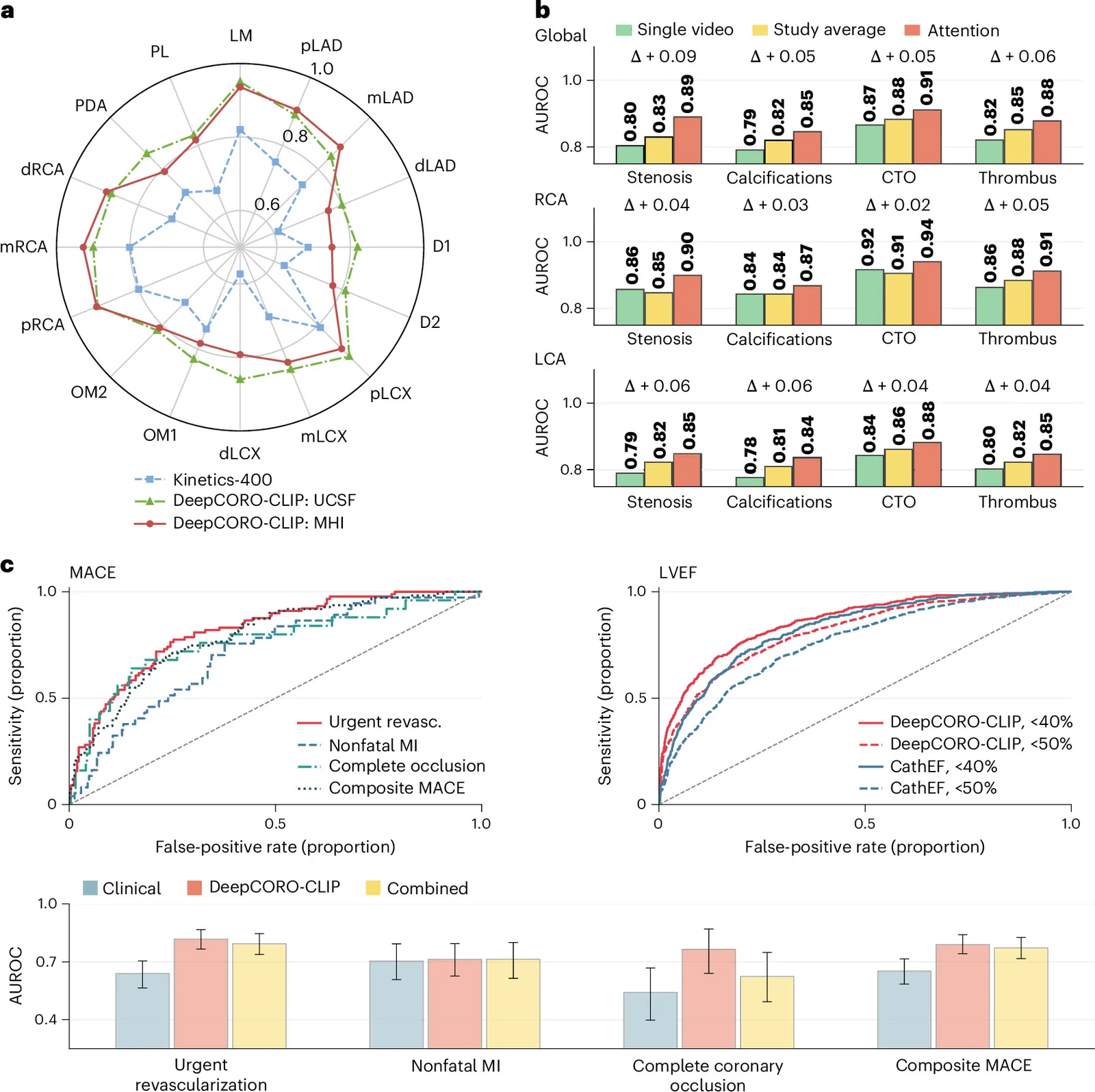
DeepCORO-CLIP performance for stenosis detection, ablation studies and transfer learning. **a**, Per-segment stenosis detection. Each value is the AUROC point estimate for detection of ≥70% stenosis in each of the 18 coronary segments, comparing the Kinetics-400 baseline and DeepCORO-CLIP pretraining in the MHI internal held-out test set (*n* = 4,828 studies) and DeepCORO-CLIP in the UCSF external validation set (*n* = 4,249 studies); no statistical test was applied. **b**, Multiview ablation experiments. Each value is the micro-averaged AUROC point estimate across all coronary segments for stenosis, calcification, CTO and thrombus detection, comparing single-video inference, study-level averaging and multivideo attention pooling in the MHI internal held-out test set (*n* = 4,828 studies). Delta values are the absolute AUROC difference from the single-video baseline. Bottom graphs show the same comparison within the RCA and LCA territories. No statistical test was applied. **c**, Transfer learning. Left: receiver operating characteristic curves for DeepCORO-CLIP prediction of 1-year MACE and its components in the held-out MACE test set (*n* = 350 patients; events: urgent revascularization, *n* = 89; nonfatal MI, *n* = 37; complete coronary occlusion, *n* = 25; composite MACE, *n* = 111). Right: receiver operating characteristic curves for LVEF estimation (classification of reduced LVEF) by DeepCORO-CLIP compared with CathEF in the MHI internal LVEF test set (*n* = 2,530 studies). Bottom: AUROC of the clinical-baseline logistic-regression model, DeepCORO-CLIP alone and the combined model for each MACE end point in the same 350-patient test cohort (patient-level fivefold grouped cross-validation); bars are AUROC point estimates (the measure of center for each error bar), and error bars are 95% CIs from 1,000 bootstrap iterations. No formal hypothesis test was applied to the bar chart; differences between models are read from the confidence intervals. D2, diagonal branch 2; OM, obtuse marginal; EF, ejection fraction. Source data

**Extended Data Fig. 3:**
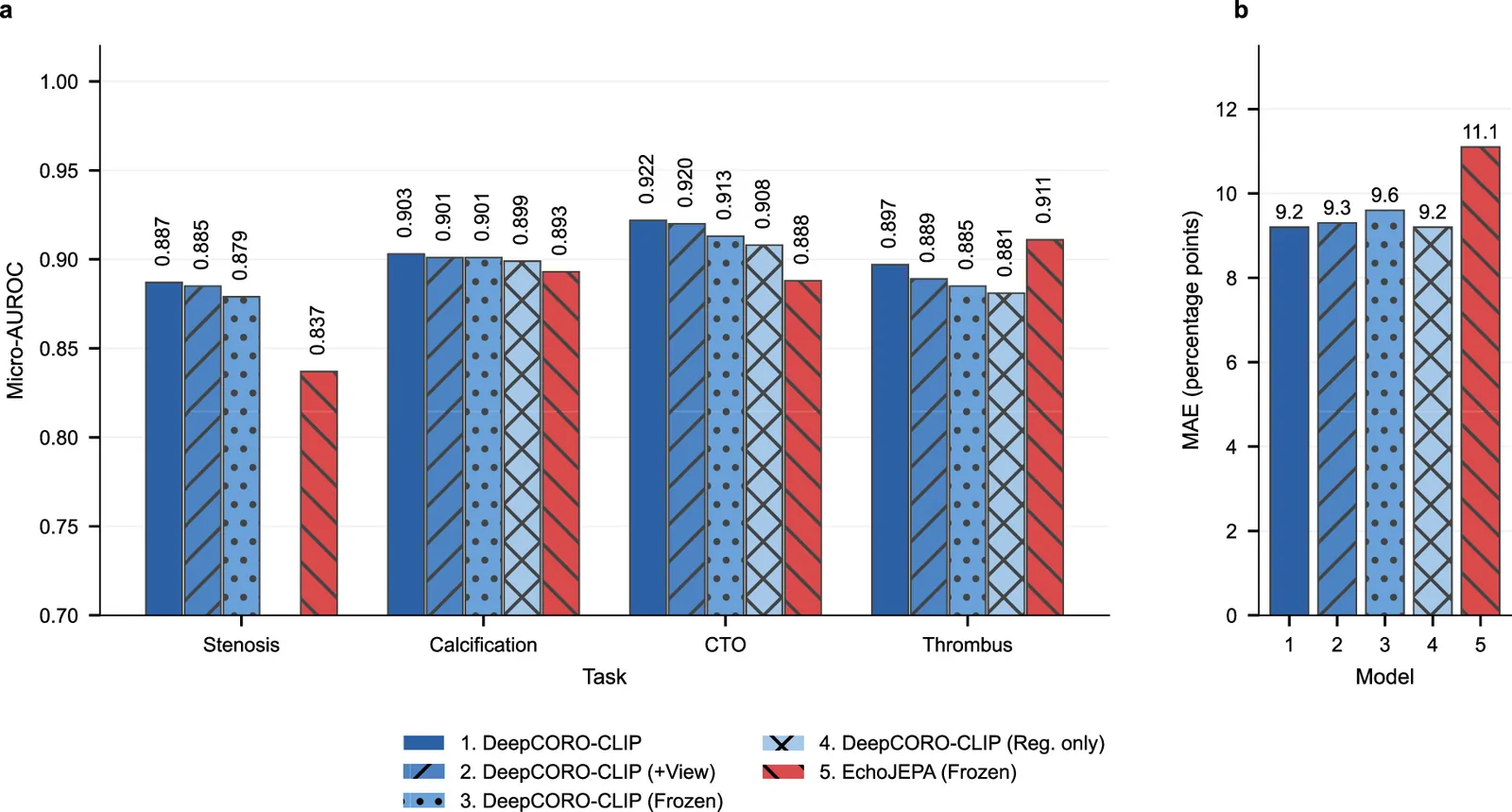
Performance across training objectives and architectures. Training objectives and architectures. n = 4,828 studies and 18 coronary segments. Each bar represents a single model evaluation. **a**, Micro-AUROC (dimensionless). No stenosis-classification result is shown for the regression-only configuration. **b**, Stenosis mean absolute error (percentage points). Model numbers correspond to the colour and pattern key. Error bars and statistical tests are not shown. Source data

Domain-specific contrastive pretraining outperformed a Kinetics-400 Multiscale Vision Transformer (MViT) linear probe across all tasks, with the largest gains for rare phenotypes (stenosis ≥70%, AUROC 0.77 to 0.89, area under the precision-recall curve (AUPRC) 0.29 to 0.52; calcification AUROC 0.77 to 0.90; CTO 0.80 to 0.92; thrombus AUPRC 0.01 to 0.23; MAE 10.4% to 9.2%; Table 2 versus Supplementary Table 11). Multivideo attention aggregation improved AUROC over single-video inference and study-level averaging (Fig. 2). Territory-level analysis (*n* = 4,784) ranged from AUROC 0.94 (left main) to 0.82 (LAD), with distal/branch vessels at 0.83; calibration slopes were 0.60 to 0.74, and distal-vessel sensitivity collapsed to 0.04 at *P* ≥ 0.5 despite specificity of >0.99 which was the dominant residual failure mode. The primary DeepCORO-CLIP configuration performed best across variants (micro-AUROC 0.89, 0.90, 0.92 and 0.90 for stenosis, calcification, CTO and thrombus; MAE 9.2; Extended Data Fig. 3): view-position embeddings gave no benefit, regression-only training slightly reduced classification while preserving MAE, and a frozen encoder remained competitive (micro-AUROC 0.88–0.91; MAE 9.6), indicating the pretrained representations are discriminative without fine-tuning. Comparing the two pretraining objectives with both encoders held frozen so that representation quality is isolated from fine-tuning capacity, the contrastively pretrained encoder outperformed a domain-adapted V-JEPA 2 encoder (EchoJEPA) on stenosis (micro-AUROC 0.88 versus 0.84), calcification (0.90 versus 0.89) and CTO (0.91 versus 0.88), while EchoJEPA was higher for thrombus (0.92 versus 0.90).

Performance was evaluated across age and biological sex subgroups (Table 3). For stenosis ≥70%, women showed higher AUROC than men (0.91 versus 0.87), with similar patterns for CTO (0.95 versus 0.91) and thrombus (0.91 versus 0.89). Stenosis AUROC decreased with age, from 0.92 (≤50 years) to 0.86 (≥80 years). MAE was lower in women (7.0%) than in men (10.7%) and rose with age from 5.8% (≤50 years) to 11.4% (≥80 years). AUROC was at least 0.86 across all subgroups and tasks. Performance did not differ across vendors (Supplementary Table 7).

**Table 3:** Lesion detection and stenosis regression performance by sex and age subgroup, RCA and LCA territories

Lesion detection metrics						
Subgroup	Prevalence/studies, *n* (%)	Prevalence/lesions (%)	AUROC^a^ (95% CI)	AUPRC^a^ (95% CI)	Sensitivity^a^ (95% CI)	Specificity^a^ (95% CI)
**Stenosis** **≥** **70%**						
Men	3,149/4,828 (65.2%)	5,637/56,682 (9.94%)	0.87 (0.87–0.88)	0.52 (0.50–0.53)	0.82 (0.81–0.83)	0.74 (0.74–0.75)
Women	1,559/4,828 (32.3%)	1,607/28,062 (5.73%)	0.91 (0.90–0.92)	0.52 (0.49–0.54)	0.85 (0.83–0.87)	0.81 (0.81–0.82)
Age ≤50 years	401/4,828 (8.3%)	347/7,218 (4.81%)	0.92 (0.91–0.93)	0.48 (0.42–0.53)	0.87 (0.84–0.91)	0.83 (0.82–0.84)
Age 51–60 years	971/4,828 (20.1%)	1,228/17,478 (7.03%)	0.90 (0.89–0.90)	0.53 (0.50–0.56)	0.83 (0.81–0.85)	0.79 (0.78–0.80)
Age 61–70 years	1,471/4,828 (30.5%)	2,257/26,478 (8.52%)	0.89 (0.88–0.89)	0.51 (0.49–0.53)	0.87 (0.86–0.89)	0.73 (0.73–0.74)
Age 71–80 years	1,435/4,828 (29.7%)	2,424/25,830 (9.38%)	0.88 (0.87–0.88)	0.52 (0.50–0.54)	0.80 (0.79–0.82)	0.78 (0.77–0.78)
Age ≥80 years	550/4,828 (11.4%)	1,056/9,900 (10.67%)	0.86 (0.85–0.87)	0.52 (0.49–0.55)	0.74 (0.72–0.77)	0.80 (0.80–0.81)
**Calcification**						
Men	3,149/4,828 (65.2%)	1,251/56,682 (2.21%)	0.89 (0.88–0.90)	0.18 (0.17–0.20)	0.84 (0.82–0.86)	0.80 (0.80–0.80)
Women	1,559/4,828 (32.3%)	460/28,062 (1.64%)	0.92 (0.91–0.93)	0.19 (0.16–0.23)	0.93 (0.91–0.96)	0.76 (0.75–0.76)
Age ≤50 years	401/4,828 (8.3%)	27/7,218 (0.37%)	0.92 (0.87–0.96)	0.15 (0.05–0.30)	0.82 (0.67–0.96)	0.90 (0.89–0.91)
Age 51–60 years	971/4,828 (20.1%)	155/17,478 (0.89%)	0.91 (0.90–0.93)	0.14 (0.10–0.19)	0.88 (0.82–0.93)	0.78 (0.78–0.79)
Age 61–70 years	1,471/4,828 (30.5%)	445/26,478 (1.68%)	0.89 (0.88–0.91)	0.15 (0.12–0.18)	0.86 (0.82–0.89)	0.78 (0.77–0.78)
Age 71–80 years	1,435/4,828 (29.7%)	743/25,830 (2.88%)	0.89 (0.88–0.90)	0.22 (0.19–0.24)	0.85 (0.82–0.87)	0.80 (0.79–0.80)
Age ≥80 years	550/4,828 (11.4%)	354/9,900 (3.58%)	0.87 (0.85–0.89)	0.22 (0.19–0.26)	0.83 (0.79–0.86)	0.76 (0.76–0.77)
**Thrombus**						
Men	3,149/4,828 (65.2%)	156/56,682 (0.28%)	0.89 (0.86–0.92)	0.19 (0.13–0.27)	0.72 (0.65–0.79)	0.92 (0.91–0.92)
Women	1,559/4,828 (32.3%)	55/28,062 (0.20%)	0.91 (0.85–0.96)	0.33 (0.19–0.46)	0.73 (0.60–0.84)	0.97 (0.97–0.97)
Age ≤50 years	401/4,828 (8.3%)	27/7,218 (0.37%)	0.91 (0.84–0.97)	0.25 (0.10–0.45)	0.81 (0.65–0.96)	0.92 (0.91–0.93)
Age 51–60 years	971/4,828 (20.1%)	62/17,478 (0.35%)	0.92 (0.88–0.96)	0.32 (0.21–0.45)	0.79 (0.69–0.89)	0.94 (0.93–0.94)
Age 61–70 years	1,471/4,828 (30.5%)	69/26,478 (0.26%)	0.88 (0.83–0.93)	0.24 (0.14–0.34)	0.73 (0.62–0.83)	0.92 (0.91–0.92)
Age 71–80 years	1,435/4,828 (29.7%)	51/25,830 (0.20%)	0.89 (0.83–0.94)	0.18 (0.08–0.30)	0.67 (0.53–0.80)	0.96 (0.96–0.96)
Age ≥80 years	550/4,828 (11.4%)	10/9,900 (0.10%)	0.89 (0.79–0.98)	0.25 (0.02–0.59)	0.69 (0.38–1.00)	0.95 (0.95–0.96)
**CTO**						
Men	3,149/4,828 (65.2%)	430/56,682 (0.76%)	0.91 (0.90–0.92)	0.18 (0.15–0.22)	0.85 (0.82–0.88)	0.83 (0.83–0.83)
Women	1,559/4,828 (32.3%)	119/28,062 (0.42%)	0.95 (0.93–0.96)	0.19 (0.13–0.25)	0.95 (0.91–0.98)	0.79 (0.79–0.80)
Age ≤50 years	401/4,828 (8.3%)	21/7,218 (0.29%)	0.96 (0.94–0.98)	0.18 (0.06–0.34)	0.91 (0.76–1.00)	0.91 (0.91–0.92)
Age 51–60 years	971/4,828 (20.1%)	103/17,478 (0.59%)	0.93 (0.91–0.95)	0.23 (0.15–0.31)	0.91 (0.85–0.96)	0.81 (0.80–0.82)
Age 61–70 years	1,471/4,828 (30.5%)	176/26,478 (0.66%)	0.92 (0.90–0.94)	0.18 (0.13–0.24)	0.82 (0.76–0.88)	0.89 (0.88–0.89)
Age 71–80 years	1,435/4,828 (29.7%)	164/25,830 (0.63%)	0.93 (0.90–0.95)	0.20 (0.14–0.26)	0.83 (0.77–0.88)	0.89 (0.88–0.89)
Age ≥80 years	550/4,828 (11.4%)	92/9,900 (0.93%)	0.88 (0.83–0.92)	0.17 (0.11–0.25)	0.82 (0.74–0.90)	0.82 (0.81–0.83)

Stenosis regression metrics			
Subgroup	Studies, *n* (%)	MAE (95% CI)	Pearson (95% CI)
Men	3,149/4,828 (65.2%)	10.7 (10.5–10.9)	0.51 (0.50–0.52)
Women	1,559/4,828 (32.3%)	7.0 (6.8–7.2)	0.55 (0.53–0.57)
Age ≤50 years	401/4,828 (8.3%)	5.8 (5.4–6.2)	0.51 (0.47–0.55)
Age 51–60 years	971/4,828 (20.1%)	8.1 (7.8–8.4)	0.53 (0.51–0.55)
Age 61–70 years	1,471/4,828 (30.5%)	9.5 (9.3–9.8)	0.51 (0.50–0.53)
Age 71–80 years	1,435/4,828 (29.7%)	10.2 (10.0–10.5)	0.52 (0.51–0.54)
Age >80 years	550/4,828 (11.4%)	11.4 (10.9–11.8)	0.53 (0.50–0.55)

^a^AUROC, AUPRC, sensitivity and specificity point estimates with 95% CIs from 1,000 bootstrap iterations; sensitivity and specificity use the Youden index. The top reports prevalence, AUROC and AUPRC with 95% CIs for stenosis ≥ 70%, CTO, thrombus and calcification; the bottom reports prevalence, MAE and Pearson correlation with 95% CIs for continuous stenosis. Both metrics use the same subgroup rows. Values are point estimates with 95% CIs from 1,000 bootstrap iterations in the internal held-out test set (*n* = 4,828 studies); no hypothesis test is reported in this table.

Studies, *n* (%), number and percentage of studies in each demographic group; prevalence/lesion, number of lesions divided by the total number of arterial segments within each demographic group; Pearson, Pearson correlation coefficient.

### Transfer learning

For MACE prediction, DeepCORO-CLIP was fine-tuned on 1,047 patients and then evaluated on 350 patients with suspected or confirmed CAD, followed up to 1 year (median 3.5 months, interquartile range 1.3 to 6.5 months); MACE occurred in 111 patients (31.7%). The model achieved an AUROC of 0.79 (95% CI 0.74 to 0.84) for composite MACE. Risk stratification separated three groups (log-rank *P* < 0.001), with hazard ratios of 5.6 for the intermediate-risk group and 18.6 for the high-risk group relative to the low-risk reference group. Discrimination for individual and composite end points is summarized in Table 4 and Fig. 2c. DeepCORO-CLIP outperformed the clinical baseline logistic-regression model on every end point with sufficient events. For urgent revascularization (*n* = 89), AUROC was 0.82 for DeepCORO-CLIP versus 0.64 for the clinical baseline; for nonfatal myocardial infarction (MI; *n* = 37), 0.71 versus 0.70; and for the composite core-events end point (*n* = 111), 0.79 versus 0.65. Adding clinical variables to the DeepCORO-CLIP risk score did not improve discrimination, with the combined model comparable to or slightly below DeepCORO-CLIP alone (urgent revascularization 0.79, nonfatal MI 0.71, composite 0.77).

**Table 4:** Classification performance for MACE prediction (test set, *n* = 350)

MACE label	*n* (abnormal/total)	AUROC (95% CI)	AUPRC (95% CI)	Sensitivity (95% CI)	Specificity (95% CI)
Urgent revascularization	89/350 (25.4%)	0.82 (0.77–0.87)	0.60 (0.50–0.71)	0.78 (0.67–0.87)	0.75 (0.66–0.84)
Nonfatal MI	37/350 (10.5%)	0.71 (0.63–0.80)	0.22 (0.14–0.34)	0.76 (0.56–0.95)	0.62 (0.43–0.86)
Complete coronary occlusion	25/350 (7.1%)	0.77 (0.64–0.87)	0.27 (0.15–0.44)	0.68 (0.48–0.89)	0.82 (0.63–0.92)
Composite MACE	111/350 (31.7%)	0.79 (0.74–0.84)	0.65 (0.56–0.73)	0.71 (0.62–0.92)	0.76 (0.53–0.84)

Classification performance for 1-year MACE prediction in the held-out test set (*n* = 350 patients; 89 urgent revascularization, 37 nonfatal MI, 25 complete coronary occlusion and 111 composite events). Values are point estimates with 95% CIs from 1,000 bootstrap iterations; sensitivity and specificity are reported at the Youden index. The risk-group comparison reported in the ‘Results’ used the two-sided log-rank test; no hypothesis test is reported in this table.

*n*, number (abnormal/total).

We also fine-tuned the encoder to predict LVEF. On the Montreal Heart Institute (MHI) internal test set (*n* = 2,530 studies), DeepCORO-CLIP outperformed the earlier CathEF (a previously published video-based AI model for estimating left ventricular ejection fraction from coronary angiograms) baseline^9^ across all regression and classification metrics (Supplementary Table 12 and Fig. 2c). For continuous LVEF, DeepCORO-CLIP achieved MAE 7.3% (95% CI 7.0 to 7.5) versus 8.8% (8.6 to 9.0) for CathEF and Pearson *r* 0.65 (0.62 to 0.68) versus 0.50 (0.47 to 0.52). In a sensitivity analysis with progressively tighter windows, LVEF estimation was stable: MAE was 7.34%, 7.32% and 7.30%, and Pearson *r* was 0.63, 0.64 and 0.65 for windows of ≤7 days (*n* = 2,055), ≤14 days (*n* = 2,291) and ≤30 days (*n* = 2,530), supporting the 30-day pairing used in the primary analysis^9^.

### Embedding analyses

**Extended Data Fig. 4:**
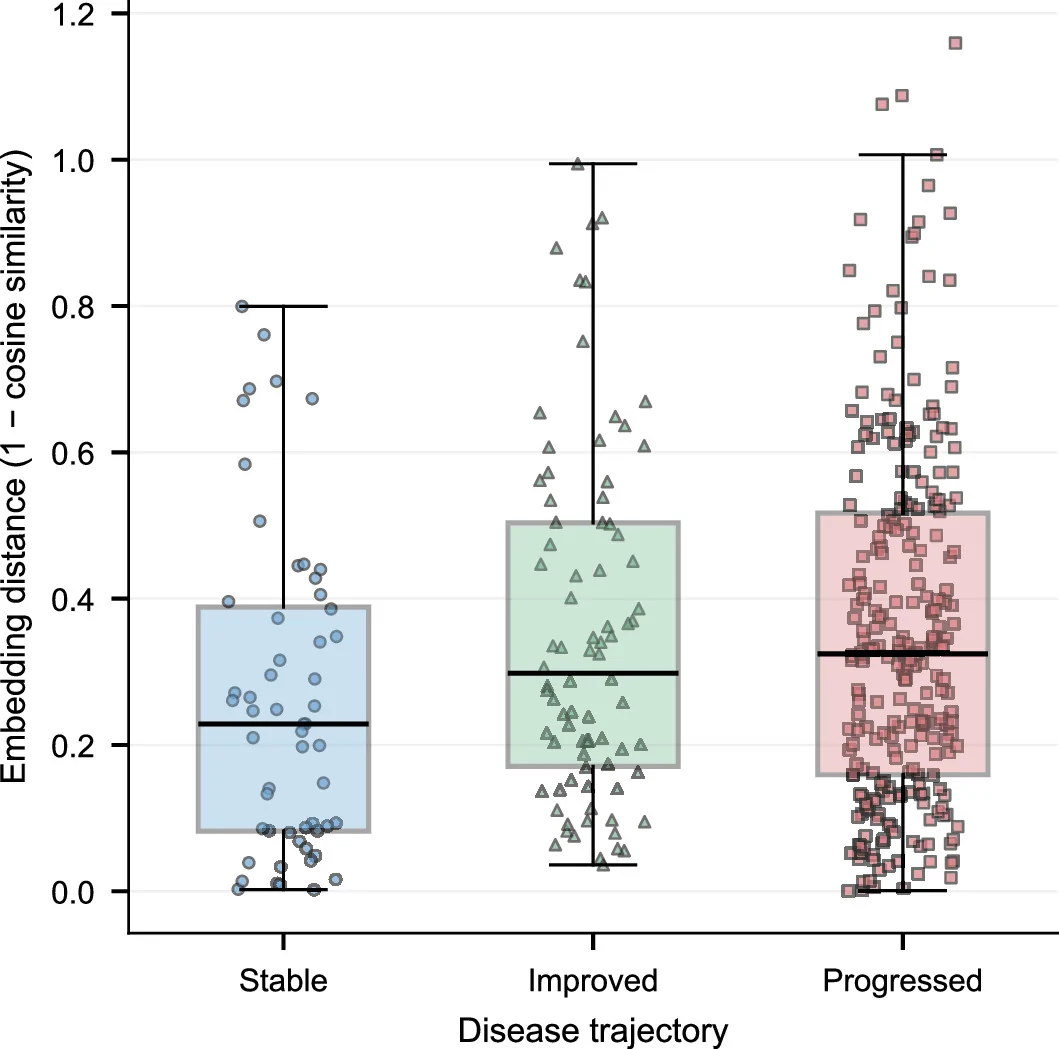
Longitudinal embedding distance by disease trajectory. Longitudinal embedding distance. n = 401 consecutive study pairs from 352 patients: stable, n = 56; improved, n = 82; progressed, n = 263. Boxes show the median and interquartile range; whiskers extend to the most extreme observation within 1.5 times the interquartile range. Points show all study pairs, horizontally jittered. Two-sided independent two-sample t-tests with equal variances gave P = 0.02 for stable versus improved and P = 0.008 for stable versus progressed. Post-PCI embeddings represent prior studies when PCI was performed. Source data

To evaluate whether DeepCORO-CLIP embeddings encode clinically meaningful representations, we compared study-level embedding distances across 401 consecutive catheterization pairs from 352 patients in the test set. Disease trajectory was classified as stable (*n* = 56, 14.0%), improved (*n* = 82, 20.4%) or progressed (*n* = 263, 65.6%). Stable pairs showed the lowest embedding distance (0.26; 95% CI 0.20 to 0.32), while both improved (0.35; 95% CI 0.30 to 0.40; *P* = 0.02) and progressed pairs (0.35; 95% CI 0.32 to 0.38; *P* = 0.008; Extended Data Fig. 4) showed significantly greater distances. The comparable distances in improved and progressed groups indicate sensitivity to anatomical change in either direction.

### Core laboratory adjudication

To assess model accuracy against expert consensus, we performed core laboratory adjudication on 662 studies comprising 965 lesions, with balanced representation of LCA (332 studies, 476 lesions) and RCA (330 studies, 489 lesions) disease (Supplementary Table 13). Interreader agreement between the two primary readers was substantial (*κ* = 0.76). At the study level, DeepCORO-CLIP showed notably lower error against adjudicated values (MAE 13.6%, 95% CI 12.5 to 15.2) compared with clinical reports (MAE 19.0%, 95% CI 17.6 to 20.5), with stronger correlation to adjudication (Pearson *r* 0.64, 95% CI 0.58 to 0.70) than to clinical reports (*r* 0.56, 95% CI 0.49 to 0.62). This pattern was consistent across both LCA and RCA territories. The weaker agreement with clinical reports reflected systematic under-call in the reports themselves: relative to core-lab adjudication, report-derived labels were specific (0.92) but insensitive (0.47), with only moderate overall agreement (Cohen’s *κ* = 0.43; Supplementary Table 13), missing a meaningful fraction of quantitative coronary angiography (QCA)-positive lesions, predominantly intermediate and severe stenoses. DeepCORO-CLIP also outperformed DeepCORO at the study level, achieving lower MAE against adjudication (13.6% versus 19.1%) despite comparable correlation coefficients (0.64 versus 0.68).

### Error analysis

In the test set of 4,828 diagnostic catheterizations, DeepCORO-CLIP correctly identified at least one treated vessel as abnormal in 85.8% of 1,736 patients who underwent PCI; missed cases were predominantly single-vessel disease (70.9%) with lower anatomical complexity by the SYNergy between PCI with TAXus and cardiac surgery (SYNTAX) score (mean 6.4 versus 11.1). Of 2,755 vessel-level PCI instances, 424 (15.4%) were performed on vessels with <70% angiographic stenosis; DeepCORO-CLIP classified 124 of these (29.2%) as normal, including 37 of 82 vessels (45.1%) with <30% stenosis. Among 3,092 patients who did not undergo PCI, 51.7% were classified as affected, although most (67.7%) had confirmed ≥70% stenosis managed with surgical revascularization or medical therapy. Only 516 (16.7%) were true false positives without obstructive stenosis; 128 of these had borderline 50% to 69% disease, leaving 343 unexplained false positives (11.1%). Of 1,493 patients classified as unaffected, 94.0% truly had no obstructive stenosis, and only 9 (0.6%) had multivessel disease. False negatives were predominantly localized to distal and branch segments (52.8% of 89 patient-level misses). The negative predictive value (NPV) exceeded 98% across all segment categories, indicating that a normal DeepCORO-CLIP prediction reliably excluded obstructive CAD.

### Precision–sensitivity trade-off of the deployed threshold

**Extended Data Fig. 5:**
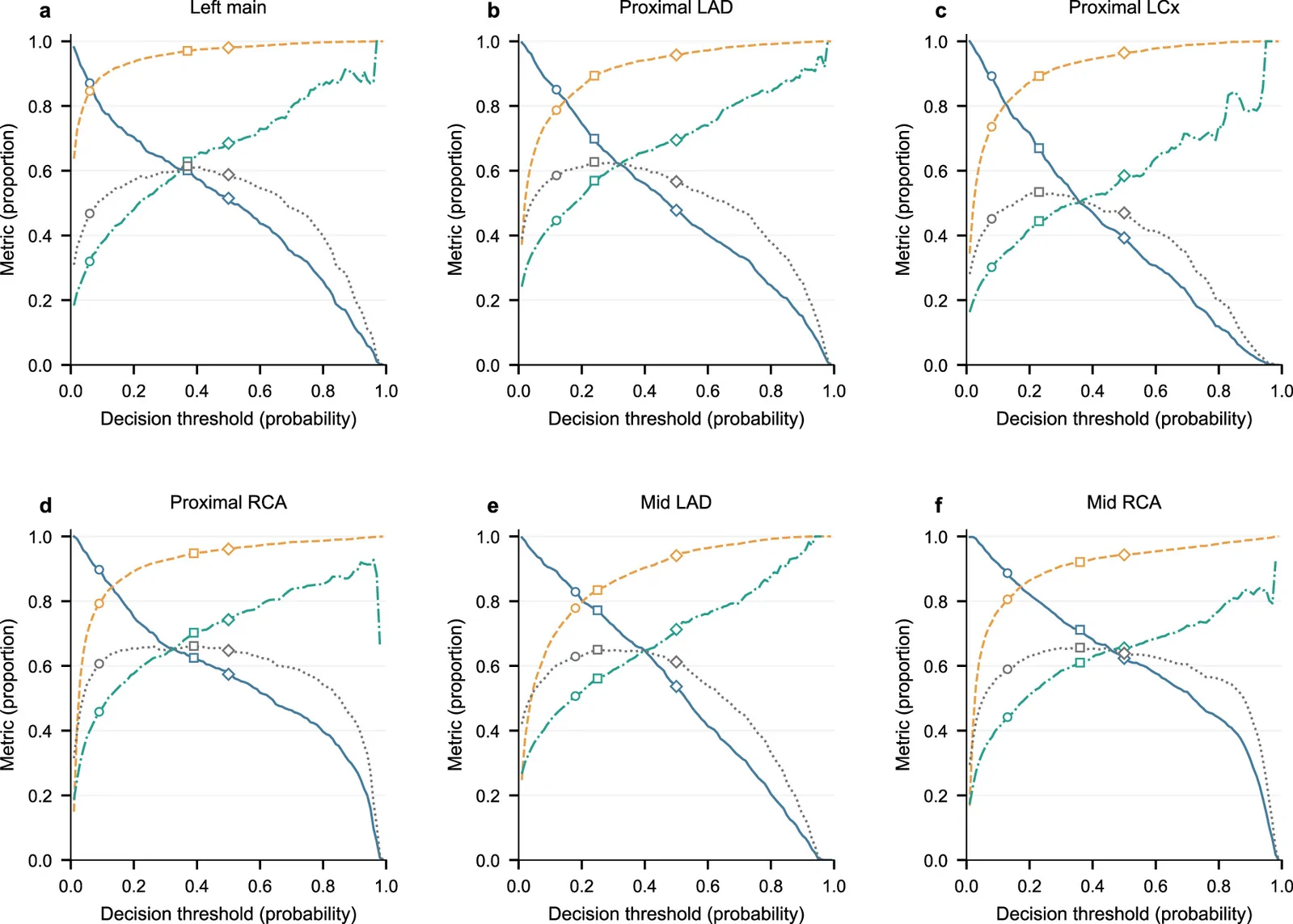
Precision and sensitivity across decision thresholds. Precision-sensitivity trade-off. Panels **a**–**f** show the left main, proximal LAD, proximal LCx, proximal RCA, mid LAD and mid RCA, respectively. Each panel plots sensitivity, specificity, positive predictive value and F1 across decision thresholds for that coronary segment. n = 4,828 studies. Sensitivity, solid blue; specificity, dashed orange; positive predictive value, dash-dot teal; F1, dotted grey. Thresholds range from 0.01 to 0.99 in increments of 0.01. Open diamonds, squares and circles mark the 0.50, maximum-F1 and Youden thresholds, respectively. Curves are point estimates; confidence bands are not shown. Source Data and Supplementary Table 7 provide 95% confidence intervals from 1,000 bootstrap iterations at marked operating points. Obstructive disease is defined as left main stenosis ≥50% and other-segment stenosis ≥70%. Threshold optimization and evaluation use the same test set. Source data

The deployed report generator calls a segment abnormal at a fixed probability of 0.50. In the six segments that accounted for the reader-study misses (left main, proximal LAD, proximal LCX, proximal RCA, mid-LAD and mid-RCA; prevalence 7.7% to 21.5%), that threshold favored precision: sensitivity 0.39 to 0.62 with positive predictive value (PPV) 0.58 to 0.74 and specificity 0.94 to 0.98. At the balanced (Youden) point (thresholds 0.06 to 0.18), sensitivity rose to 0.83 to 0.90, while PPV fell to 0.30 to 0.51, with the max-F1 threshold (0.23 to 0.39) between the two (Extended Data Fig. 5 and Supplementary Table 14). The trade-off was steepest where prevalence was lowest: in the left main (7.7%), moving to the balanced threshold raised sensitivity from 0.51 to 0.87 but cut PPV from 0.68 to 0.32, so roughly two of every three flags would be false. The proximal misses therefore reflect the deployed threshold, not the model’s discrimination.

### Cross-institutional validation

In our external validation dataset, we used a large language model (LLM) to match stenoses from free-text report to the correct segment. To verify the accuracy of these LLM-derived labels, a severity-stratified random sample of 236 reports (4,248 segment labels) was independently re-extracted by two interventional cardiologists. Agreement between the cardiologists and the LLM extraction was near perfect for the clinically decisive obstructive call (ranges among vessel segments: Cohen’s *κ* = 0.91 to 0.93; sensitivity 0.96 to 0.98, specificity 0.99) and for continuous severity (intraclass correlation 0.92, MAE approximately 2.7 percentage points) with only 1.6% of labels disagreeing on the obstructive threshold (Supplementary Table 8). External validation at the University of California, San Francisco (UCSF) showed preserved discriminative performance for stenosis ≥70% detection despite differences in population characteristics and imaging protocols (Supplementary Table 15). The UCSF cohort had lower disease prevalence (42.2% with obstructive stenosis) compared with the internal validation set (59.8%). Global AUROC for stenosis ≥70% was 0.89 (95% CI 0.88 to 0.89) with sensitivity of 0.84 and specificity of 0.77. Segment-level AUROC ranged from 0.80 (distal LAD) to 0.95 (left main), with proximal segments showing strongest performance (proximal LAD, 0.89; proximal LCX, 0.92; proximal RCA, 0.92). Regression performance showed a global MAE 12.1% (95% CI 11.9 to 12.3) and Pearson *r* 0.45 (95% CI 0.44 to 0.46). Compared to internal validation, AUROC remained stable across most segments (left main, 0.95 versus 0.93; proximal RCA, 0.92 versus 0.90), while MAE increased modestly (proximal LAD, 18.8% versus 16.9%; mid-LAD, 20.9% versus 19.3%). Pearson correlations were lower externally (proximal LAD, 0.59 versus 0.61; mid-RCA, 0.58 versus 0.61).

### Prospective single-report reader validation

**Extended Data Fig. 6:**
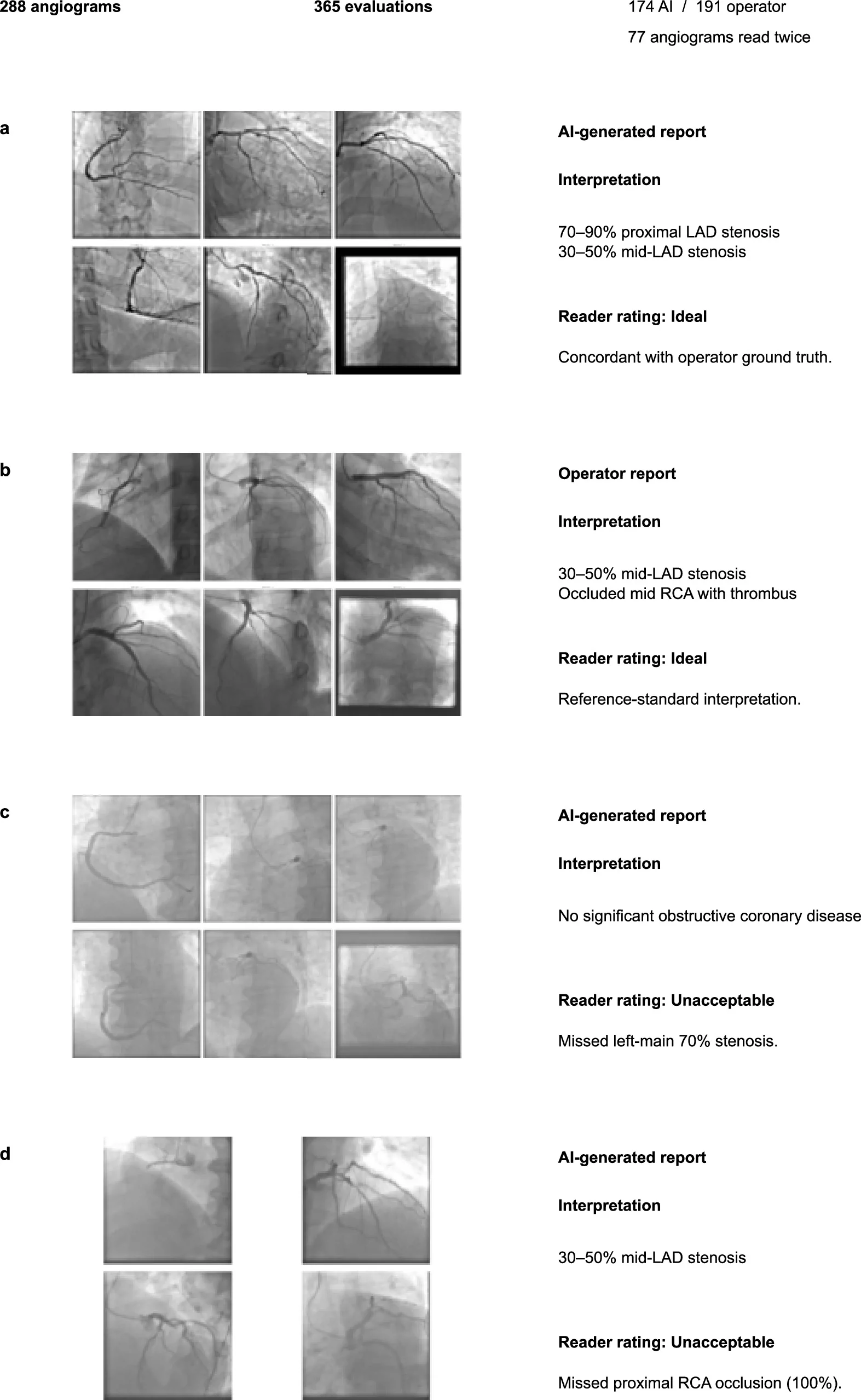
Prospective blinded reader validation. Reader-study flow and representative interpretations. n = 288 angiograms and 365 evaluations: 174 AI-generated reports and 191 operator reports; 77 angiograms were independently read twice. **a–d**, Four representative interpretations and their reader conclusions. The examples illustrate individual cases and are not a statistical sample. Source data

To assess the standalone clinical quality of the model’s text reports, we performed a prospective, blinded, single-report reader study. Report evaluations were completed by two independent readers across 288 unique angiograms, yielding 365 evaluations (174 AI, 191 operator-generated reports), of which 77 studies were read by both readers (Table 5 and Extended Data Fig. 6). AI reports were rated clinically usable (ideal or acceptable) in 76% of evaluations versus 88% for operator reports (Fisher odds ratio 0.41, *P* = 0.002) and unacceptable in 24% versus 12% (*P* = 0.007), with ideal ratings of 40% versus 46%. AI reports scored lower than operator reports across all three quality domains (clinical correctness 2.97 versus 3.37, *P* = 0.012; completeness 3.04 versus 3.48, *P* = 0.007; clarity 3.03 versus 3.36, *P* = 0.021; all confirmed by the Mann–Whitney *U*-test). The quality gap was driven by omissions rather than fabrication: a hallucinated finding was flagged no more often in AI than in operator reports (17% versus 14%; Fisher odds ratio 1.32, *P* = 0.38), whereas an unacceptable AI report was almost always attributable to a missed proximal obstructive stenosis (left main, proximal LAD or proximal RCA), the same segments in which the deployed 0.50 threshold was most precision oriented (Extended Data Fig. 5 and Supplementary Table 14). Among 77 doubly read studies, readers agreed on the usable versus unacceptable classification in 84% of cases (Cohen’s *κ* = 0.17); the low kappa despite high raw agreement reflects the strong imbalance between usable and unacceptable ratings and indicates that agreement on which specific reports were unacceptable was weak, so the acceptability estimates should be read as reader dependent. Representative interpretations spanning the quality range are shown in Extended Data Fig. 6.

**Table 5:** Prospective blinded single-report reader study: DeepCORO-CLIP versus operator reports

Outcome	DeepCORO-CLIP (*n* = 174)	Operator (*n* = 191)	Effect (95% CI)	*P*
Ideal, *n* (%)	70 (40)	88 (46)	chi-square = 10.06	0.007
Acceptable, *n* (%)	62 (36)	81 (42)		
Unacceptable, *n* (%)	42 (24)	22 (12)		
Clinically usable (ideal or acceptable), *n* (%)	132 (76)	169 (88)	OR 0.41 (0.23 to 0.72)	0.002
Clinical correctness, mean (s.d.)	2.97 (1.12)	3.37 (0.85)	Difference −0.40 (−0.70 to −0.09)	0.012 (MWU 0.027)
Completeness, mean (s.d.)	3.04 (1.15)	3.48 (0.82)	Difference −0.44 (−0.75 to −0.12)	0.007 (MWU 0.017)
Clarity, mean (s.d.)	3.03 (0.99)	3.36 (0.78)	Difference −0.34 (−0.62 to −0.06)	0.021 (MWU 0.037)
Most clinically important lesion correctly identified, *n* (%)^a^	48 (67)	80 (89)	OR 0.25 (0.11 to 0.57)	0.001
Clinically important missed finding flagged, n (%)	41 (24)	31 (16)	OR 1.59 (0.95 to 2.68)	0.088
Hallucinated finding flagged, *n* (%)	30 (17)	26 (14)	OR 1.32 (0.75 to 2.34)	0.384
Angiogram quality, mean (s.d.)	3.46 (0.83)	3.54 (0.79)	—	0.278

Two independent interventional cardiologists, masked to report source, evaluated 365 single-report presentations across 288 unique angiograms drawn from the held-out diagnostic test cohort (174 DeepCORO-CLIP reports, 191 operator reports); 77 studies were read independently by both readers. The study flow is shown in Extended Data Fig. 6. Overall acceptability was compared across its three categories with the chi-square test; binary outcomes with the Fisher exact test (odds ratios with 95% CIs); and the 1 to 4 ordinal domain scores with the Welch *t*-test, confirmed by the Mann–Whitney *U*-test. All tests are two-sided at an alpha of 0.05 without correction for multiple comparisons; the Effect column gives the chi-square statistic, odds ratio or mean difference with its 95% CI, and the *P* column gives the corresponding *P* value. The missed-finding and hallucination rows are counted over all evaluations, as an unanswered question indicates that the reader did not raise that problem; the most-important-lesion row is counted over the evaluations in which that question was answered.

^a^The most clinically important lesion row is counted only over evaluations in which that question was answered. OR, odds ratio; MWU, Mann–Whitney *U*-test.

### Deployment

**Extended Data Fig. 7:**
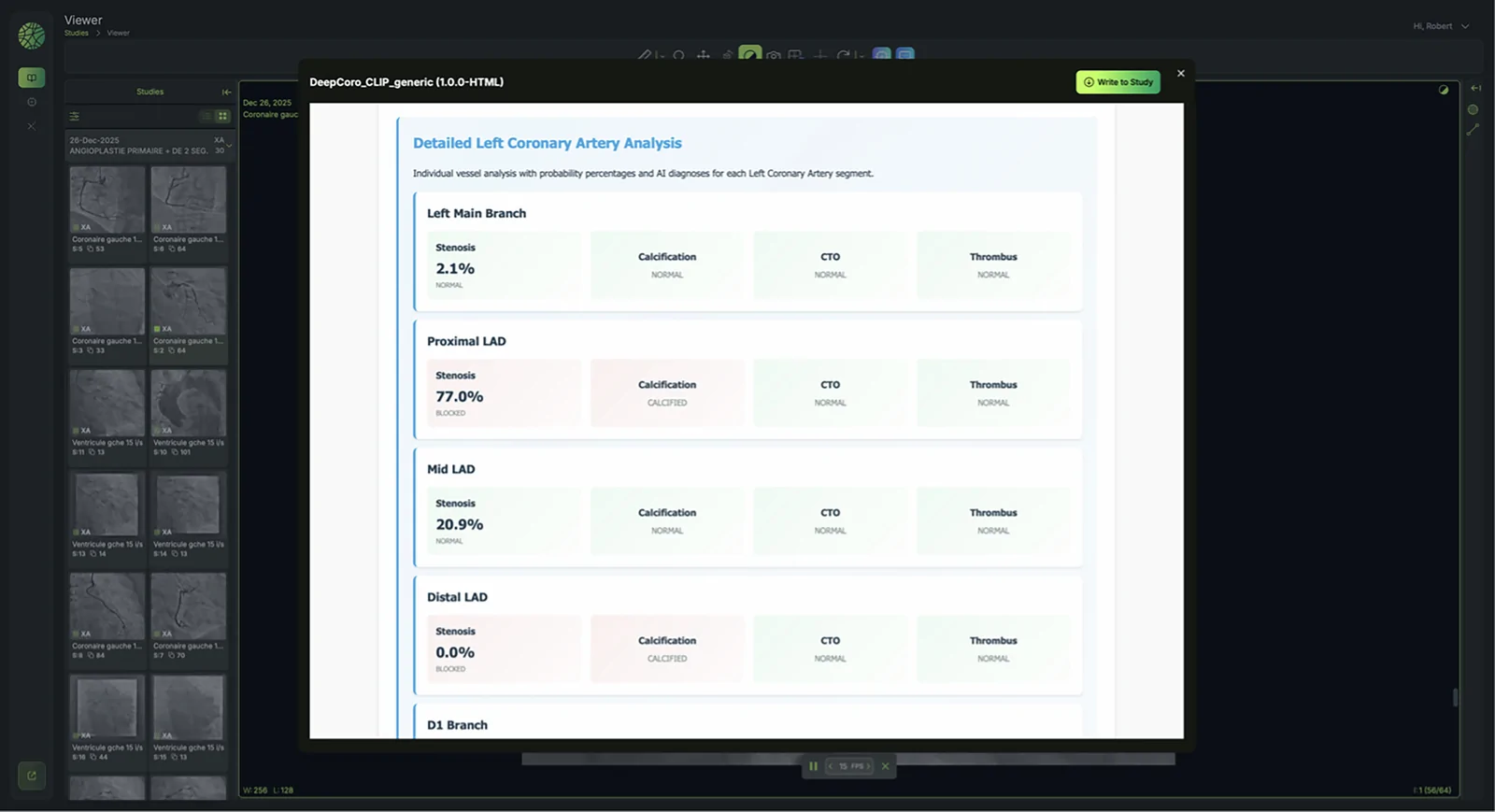
PACS AI deployment of DeepCORO-CLIP. PACS-AI deployment. Coronary-analysis output generated by DeepCORO-CLIP for an example study, displayed within the PACS viewing interface. Source data

DeepCORO-CLIP was successfully deployed using our bespoke software called PACS-AI^10^ in our catheterization laboratory workflow, enabling coronary angiography studies to stream from the catheterization lab to the picture archiving and communication system (PACS) with automated, near-real-time DeepCORO-CLIP inference (Extended Data Fig. 7). End-to-end latency, measured as wall-clock time from study receipt by the PACS-AI listener to availability of model outputs within the PACS viewing environment, was 4.2 ± 2.4 s (*n* = 24). This latency includes Digital Imaging and Communications in Medicine ingestion, video buffer construction and preprocessing (including normalization, model inference and template-based clinical report generation).

## Discussion

DeepCORO-CLIP is a multiview, video–text model for comprehensive coronary angiography interpretation. Trained on 7 years of data from one of Canada’s highest-volume catheterization centers, it integrates multiple angiographic projections through attention-based pooling for study-level assessment across a wide range of clinical tasks. For binary stenosis detection, the model achieved strong discrimination (micro-AUROC 0.89, 95% CI 0.88 to 0.89) with NPV exceeding 98%, while continuous quantification correlated moderately with reports, strongest in proximal segments. It also addresses tasks where no previous AI models exist, including CTO detection and intracoronary thrombus identification. Transfer-learning experiments suggest the representations generalize to prognostic tasks including MACE prediction and LVEF estimation, and embedding-distance analysis across serial exams detected anatomical change over time, although these applications require further validation. Our contrastive pretraining outperformed standard video encoders initialized with Kinetics-400, and multiview aggregation gave consistent gains over single-video inference and naive averaging. External validation preserved discrimination across institutions, with lower error against QCA adjudication than single-reader reports. In a prospective, blinded reader study, its automatically generated reports were clinically usable in three-quarters of studies, with the shortfall driven by conservative omissions rather than fabricated findings, supporting a human-in-the-loop reporting role while a larger multireader evaluation continues.

Our multiview approach addresses a fundamental limitation in existing coronary angiography AI systems, which predominantly rely on single-frame analysis^5^ or single-projection analysis^6^. Compared with previous coronary angiography AI systems, DeepCORO-CLIP had lower stenosis regression error than CathAI^5^ and DeepCORO^6^ (absolute differences: MAE 9.0% to 9.9% versus 17.9% to 19.1%), with additional gains when evaluated against core laboratory QCA references (MAE 13.6% versus 19.0% for clinical reports), in line with previously reported QCA percent diameter stenosis variability^11^. Our findings indicate that DeepCORO-CLIP predictions more closely approximate multireader consensus with QCA than single-reader clinical interpretations, suggesting the model captures a more generalizable assessment of stenosis severity. Previous work by Du et al.^12^ developed DeepDiscern, a multitask system for coronary angiography analysis that, like DeepCORO-CLIP, addressed lesion morphology, such as calcification, beyond stenosis quantification alone. However, DeepDiscern relied on single-frame inputs rather than video, precluding integration of temporal contrast dynamics, and used separate task-specific networks rather than a unified video–text model. External validation was not performed. DeepCORO-CLIP improves upon these limitations through video-based analysis with multiview attention pooling, vision-language contrastive pretraining enabling efficient transfer to multiple downstream tasks from a single encoder, and external validation showing cross-institutional generalizability. For comparable lesion morphology tasks, DeepCORO-CLIP achieved state-of-the-art discriminative performance (CTO AUROC 0.90 to 0.95 versus 0.76; thrombus AUROC 0.90 versus 0.78; calcification AUROC 0.90 to 0.91 versus 0.80)^12^, trained on approximately eight times more data with video rather than static frame inputs.

Our video–text contrastive learning approach marks a shift from task-specific models toward comprehensive coronary assessment. This is consistent with implementations in other medical imaging domains. In echocardiography, EchoPrime showed that multiview vision-language models trained on over 12 million video–report pairs achieved state-of-the-art performance across 23 benchmarks of cardiac form and function, surpassing task-specific and previous foundation models across five independent healthcare systems^7^. More recently, EchoJEPA^13^ showed that latent predictive architectures outperform contrastive learning in echocardiography by decoupling anatomical signal from speckle noise. However, when applied to coronary angiography and compared with both encoders held frozen, contrastive learning (DeepCORO-CLIP) outperformed the latent predictive approach (EchoJEPA) despite V-JEPA 2’s 10 times more parameters (300 versus 30 million) on stenosis, calcification and CTO detection, suggesting the optimal pretraining objective is modality dependent and that angiography’s structured visual signal favors vision-language alignment over pixel-agnostic prediction. Beyond diagnosis, the DeepCORO-CLIP encoder improved LVEF estimation from angiographic videos (MAE 7.3% versus 8.8% for CathEF)^9^, showing the learned representations transfer to functional assessment. Our 203,808 video–report pairs form one of the largest coronary angiography datasets reported, consistent with scaling benefits where larger vision-language datasets improve downstream performance. The continued improvements across all clinical tasks compared with previous work^6^ suggest that angiography interpretation especially benefits from large-scale training, given high variability in anatomical and pathological presentations. DeepCORO-CLIP achieved lower stenosis error than best human performance against QCA^2–4^, positioning it as a practical tool for generating preliminary reports, monitoring disease progression and reducing interpretive variability, particularly in single-operator settings where the absence of peer review amplifies inconsistency. Whether embedding distance can track within-patient disease progression between serial examinations remains untested. Our same-patient analysis shows only that embeddings encode patient-specific features; it does not establish that embedding displacement quantifies interval change. We frame this as a hypothesis for future work: if validated against serial QCA, embedding distance could provide an automated continuous marker of angiographic change, clinically relevant given evidence that angiographic progression carries strong prognostic value, with mortality rising stepwise as luminal narrowing advances over time^14^.

The learned representations during the pretraining phase can also be adapted to novel tasks for which no previous angiogram model was developed: DeepCORO-CLIP achieved AUROC 0.79 for composite MACE prediction comparing favorably with established risk scores requiring substantial clinical inputs. The PRAISE score, a machine learning model incorporating 25 clinical features assessed at discharge including therapeutic decisions and procedural outcomes, achieved AUROC 0.74 to 0.82 for 1-year MI prediction^15^. The age, biomarkers and clinical history–acute coronary syndrome ischemia score requires specialized biomarkers (GDF-15, NT-proBNP) not routinely available, achieving c-indices of 0.71 to 0.72 for cardiovascular death/MI^16^. Traditional clinical scores show more modest discrimination: GRACE AUROC 0.70, thrombolysis in myocardial infarction (TIMI) 0.68, PAMI 0.69 and CADILLAC 0.69 for short-term MACE following primary PCI^17^. Even large-scale machine learning approaches leveraging over 12,000 features and transformer-based tabular foundation models across 44,462 catheterizations achieved comparable or lower AUROC for MACE prediction (0.69 to 0.76) in external validation^18^. DeepCORO-CLIP derives prognostic information exclusively from the diagnostic angiogram videos without requiring patient demographics, comorbidities, biomarkers or procedural variables, enabling risk stratification at initial coronary assessment. In a sensitivity analysis, logistic regression on routinely available clinical variables (age, sex and conventional cardiovascular risk factors) alone was less accurate than DeepCORO-CLIP on the same dataset, and adding those variables to the DeepCORO-CLIP score did not improve discrimination. This suggests that most of the relevant prognostic information (disease extent, lesion severity and territorial distribution) is already encoded in the angiographic videos and captured directly by the encoder, so the clinical features were largely redundant and contributed variance rather than complementary signal at this cohort size.

We deployed DeepCORO-CLIP within a PACS-integrated inference pipeline (PACS-AI; www.pacsai.co)^10^ achieving <5-s processing, enabling near-real-time assessment during PCI planning. This gives operators immediate stenosis quantification, lesion characterization and risk stratification from the diagnostic angiogram before intervention, supporting revascularization decisions, particularly in high-volume or single-operator settings where interpretive variability is highest. Its hierarchical attention design combines intravideo temporal modeling with cross-video multiview aggregation, integrating information within and across angiographic projections. This positions DeepCORO-CLIP as a general-purpose backbone for future tasks, including automated SYNTAX scoring, preliminary report generation and longitudinal disease surveillance through serial embedding comparison. In this prospective, blinded, single-report reader study, a prospective reader-based evaluation of automatically generated text interpretations for ICA, DeepCORO-CLIP reports were clinically usable in roughly three-quarters of studies but remained inferior to expert operator reports across every quality domain. Two features temper this gap. When the model was correct, its reports matched expert quality (40% versus 46% rated ideal), and, importantly for safety, the model did not fabricate findings more often than clinicians (hallucination 17% versus 14%, *P* = 0.38); its shortfall was dominated by conservative omissions rather than invented disease, a failure profile more compatible with a human-in-the-loop workflow than confident over-reading would be. The residual gap was concentrated in one clinically important failure mode: severe proximal stenosis reported as absent. This reflects where the deployed report draws its line, not what the model can see. The report generator calls a segment abnormal only when it is fairly confident, which keeps false alarms low but lets borderline proximal lesions pass unmentioned. Loosening that threshold recovers most of the missed lesions but roughly halves the precision of what is flagged (Extended Data Fig. 5 and Supplementary Table 14). The choice is therefore a workflow question rather than a modeling one: a report read as a screening prompt, with a physician confirming every flag, can absorb the extra review burden that higher sensitivity brings; a standalone interpretation cannot. Because these thresholds were derived in the same test set in which they were evaluated, the achievable gains are optimistic and would need prospective confirmation before any threshold is fixed for clinical use. Rather than impose one, we expose the per-segment threshold as a user-configurable parameter in the released code, so each site can set, and re-derive locally, its own precision–sensitivity balance.

Several limitations warrant consideration. First, the retrospective design may not capture real-world clinical decision-making, and prospective validation is needed to assess procedural-planning impact and temporal stability. Segment-level analysis showed a proximal-to-distal gradient: DeepCORO-CLIP discriminated obstructive disease well in the left main and proximal/mid-segments (AUROC up to 0.94) but was unreliable in distal and side-branch segments, where severity correlation collapsed toward 0 (for example, distal LAD *r* = 0.01), so distal and branch estimates should be interpreted with caution. Second, patients with previous CABG were excluded, limiting applicability there. Third, external validation covered only one center; broader validation across diverse ethnic populations and healthcare systems with varying acquisition protocols remains necessary. The cohort was 66.6% men, consistent with tertiary-center referral but limiting generalizability to women, who more often present with nonobstructive disease and atypical lesion distributions; although sex-stratified AUROC remained ≥0.87, absolute calibration in women warrants prospective re-evaluation. Fourth, the study does not compare against fractional flow reserve or intravascular imaging, which could be tested in future. Fifth, video–text alignment relies on report-derived labels with inherent noise and potential misalignment with video content; robust label-noise handling was not formally addressed. For the external cohort, the reference standard derives from clinical reports rather than core-laboratory QCA; we mitigated this by comparing LLM extraction with independent re-extraction by two interventional cardiologists, with agreement on obstructive-disease labels with Cohen’s *κ* = 0.91 to 0.93 and intraclass correlation coefficient 0.92, indicating small residual noise, although a core-laboratory comparison on the external cohort awaits future work. Sixth, performance depends on adequate video quality, which may limit applicability in resource-constrained settings or for suboptimal acquisitions, although our results exceed previous literature. For MACE prediction, although discrimination exceeded clinically used models, two further limitations apply. Negative controls required documented event-free follow-up within 7 to 365 days; patients lacking follow-up were excluded rather than censored, potentially introducing selection bias and limiting generalizability. Follow-up was also short, constraining the durability of 1-year MACE estimates and precluding longer-term assessment. Finally, the reader study was conducted with two readers at a single center, included only 77 doubly read studies, and showed only slight chance-corrected agreement on report acceptability (Cohen’s *κ* = 0.17), so the 76% versus 88% usability estimate is reader dependent; in addition, the max-F1 and balanced operating points characterizing the precision–sensitivity trade-off were both derived and evaluated in the same held-out test set and are therefore optimistic. Both the acceptability estimates and the sensitivity achievable after recalibration require confirmation in a larger, multireader, prospective evaluation with prespecified thresholds. Future directions include interpretability mechanisms, additional outputs such as coronary artery segmentation and stenosis bounding-box detection, intra-procedural guidance, and integration with intravascular ultrasound and optical coherence tomography.

DeepCORO-CLIP shows that video–text contrastive pretraining on coronary angiography produces general-purpose representations that transfer effectively across diagnostic, prognostic and longitudinal monitoring tasks. By integrating multiple angiographic views into a unified embedding space, the model captures coronary anatomy and pathology at a level exceeding single-reader interpretation. With publicly released code, weights and deployment infrastructure, and a prospective reader study showing that its reports are clinically usable in 76% of evaluations and err through conservative omission rather than fabrication, DeepCORO-CLIP provides a foundation for human-in-the-loop angiographic interpretation and a building block for multimodal cardiovascular AI systems.

## Methods

### Study datasets and study population

This was a multicenter retrospective observational cohort study. The study was approved by the MHI Research Ethics Board (approval 2021-2692), which waived the requirement for written informed consent because the study involved secondary use of deidentified data. We included all patients between 2017 and 2024 who underwent ICA at the MHI based on the inclusion and exclusion criteria described below. We report the work in this paper using the Transparent Reporting of a multivariable prediction model for Individual Prognosis or Diagnosis with Artificial Intelligence guidelines^19^. Patients were eligible for inclusion if they: (1) underwent diagnostic ICA or PCI during the study period; (2) had complete angiographic video recordings with at least a video of the LCA and a video of the RCA; (3) had accompanying procedural reports with structured clinical annotations; (4) were aged 18 years or older. Patients were excluded if they had: (1) incomplete exams without at least one video of the LCA or RCA (2) previous CABG. Data were split at the patient level (ensuring no patient appears in multiple sets) into training (19,681 patients, 70%, 142,766 videos), validation (4,217 patients, 15%, 30,774 videos) and test (4,219 patients, 15%, 30,268 videos). All sensitivity analyses are performed using the same split of patients.

### Data extraction

We copied all coronary angiograms to our research environment, extracted primary and secondary angulation angles from Digital Imaging and Communications in Medicine metadata, and exported videos as MP4. We classified videos into 12 standardized angiographic views (Supplementary Table 1), consistent with established angiographic projection definitions^20^. We applied a fine-tuned X3D-M video classification model^6^ to automatically improve video selection and separate the diagnostic phase of the procedure from the interventional phase. The model predicted: (1) contrast opacification presence (binary); (2) primary anatomical structure across 12 classes (including LCA and RCA, presence of bypass grafts, catheters and other cardiovascular structures); (3) stent presence (binary); and (4) coronary dominance (right dominant, left dominant, co-dominant; Supplementary Table 2). The model was retrained for this study on 1,395 manually annotated unique angiogram studies (two cardiologists with ≥5 years’ experience each; 100% target-label consensus), split into 1,186 training, 69 validation, and 140 held-out test videos for view classification and coronary structure identification. Training was performed in a multitask setting with four classification heads, operating on clips of 72 frames resized to 256 × 256 pixels. The model showed strong performance across all tasks achieving a micro-average AUROC of 0.94 (95% CI 0.90 to 0.97) on the test set (Supplementary Tables 3 and 4).

Using this automated classification system, we categorized each video into one of three procedural phases per coronary artery based on acquisition timestamp and sequential context: diagnostic (contrast opacification without interventional equipment), interventional (guide-wire detected) or postprocedural (any video acquired after interventional equipment was first detected in a preceding image acquisition for that artery). Once interventional equipment was found in each coronary artery, all subsequent videos of that artery were classified as procedural if equipment was visible or as postprocedural if the equipment was removed. Only diagnostic videos were retained for all model training and analyses, as angioplasty modifies stenosis severity, rendering interventional and postprocedural videos unsuitable for baseline disease characterization.

### Demographics and clinical variables

Age, sex, body mass index, cardiovascular risk factors, X-ray imaging system vendor and medical history were extracted from electronic medical records and coronary angiography reports. At MHI, all coronary angiography reports are written by faculty interventional cardiologists with at least 5 years of experience each performing ≥1,500 coronary angiographies annually. Structured reports provided annotations for the downstream tasks. Coronary stenosis severity is determined using a two-step validation process: initial assessment by X-ray technologists with ≥5 years of catheterization laboratory experience, followed by review and modification by interventional cardiologists. Only the interventional cardiologist assessment is saved as part of the medical record. QCA is systematically used in our lab for intermediate lesions (40–60% diameter stenosis), while all other lesions are graded by visual estimation. Obstructive stenosis is defined as ≥70% diameter reduction (≥50% for left main). Coronary calcifications are graded as mild (visible in one projection), moderate (visible in two projections involving both sides of the arterial wall) or severe (dense calcification throughout the lesion with tram-track appearance); for binary classification, mild-to-severe calcification was considered positive, while absent calcification was considered negative. Intracoronary thrombus was identified as a discrete intraluminal filling defect surrounded by contrast on three sides or persistent contrast staining following clearance. CTO was defined as complete vessel obstruction with TIMI grade 0 flow and estimated duration ≥3 months based on clinical history or angiographic features (presence of collaterals). All segments following the 16-segment SYNTAX definition were graded for stenosis, CTO, thrombus and calcification^21^ (Supplementary Table 5). Mid- and distal left circumflex segments as well as marginal 1 and ramus intermedius were labeled separately in our database and therefore analyzed as distinct segments, yielding 18 total coronary segments rather than the standard 16.

#### Algorithm development

We trained the model in two phases: contrastive pretraining and then the model fine-tuning for downstream tasks (Fig. 1). In the first phase, the video encoder was pretrained with video–text contrastive learning, aligning each angiographic video with its paired report text to learn general-purpose coronary representations without task-specific labels. In the second phase, these representations were adapted to the downstream clinical tasks by fine-tuning the encoder together with attention-based multivideo pooling and task-specific prediction heads for study-level diagnostic assessment.

**Extended Data Fig. 8:**
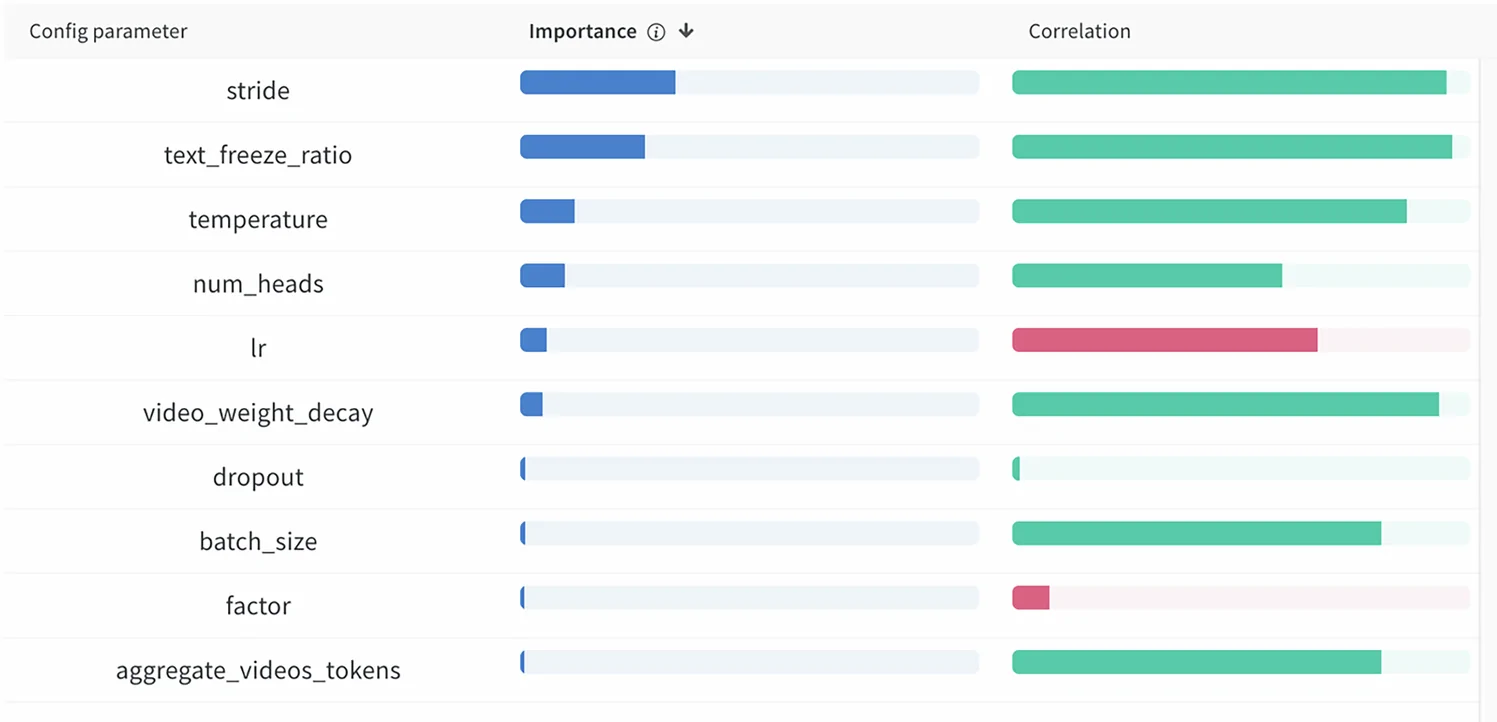
Hyperparameter sweep for contrastive video encoder training. Legend: Systematic hyperparameter optimization across experimental runs identified temporal stride, text encoder freeze ratio, and contrastive temperature as the most influential parameters for model performance. Feature importance scores (left, blue) quantify the relative impact of each hyperparameter on validation loss, with stride showing the highest importance followed by text encoder freezing strategy. Correlation analysis (right) reveals the directional relationship between each parameter and validation loss, where positive correlations (teal) indicate parameter increases that degrade performance and negative correlations (pink) indicate parameter increases that improve performance. Learning rate exhibited strong negative correlation with validation loss, indicating that lower learning rates within the explored range (5.8 × 10^−6^ to 8.0 × 10^−5^) improved contrastive alignment. Conversely, temporal stride, text freeze ratio, and temperature showed positive correlations, suggesting optimal performance at lower values within their respective ranges. Minor importance parameters including dropout, batch size, and video token aggregation strategy showed minimal influence on final model performance. Abbreviations: lr, learning rate; num_heads: number of attention heads in multi-instance learning module.

Full model architecture, contrastive pretraining, task-specific fine-tuning, encoder probing and visualization, and the supervised MViT baseline comparator are detailed in Supplementary Information; the hyperparameter search ranges and sweeps for both training phases are reported in Supplementary Tables 9 and 10 and Extended Data Fig. 8.

### Transfer learning

To evaluate transfer learning, we fine-tuned the pretrained DeepCORO-CLIP encoder for MACE prediction in the subset of patients with longitudinal follow-up ≥7 days after angiogram. Eighty percent of the layers of the video encoder (12/16) were frozen, with remaining layers fine-tuned using RAdam optimizer with cosine-warmup schedule over 50 epochs. For each study, up to 10 angiographic views were aggregated via multihead attention pooling, and separate binary classification heads were trained for each outcome. A subset of patients with documented follow-up was randomly sampled from the catheterization cohort linked to hospital administrative records (2017 to 2024). The index date was defined as catheterization date, with events within 7 days excluded to avoid periprocedural complications. Data were partitioned at patient level into training (60%, *n* = 1,047), validation (20%, *n* = 349) and test (20%, *n* = 350) sets using stratified sampling. Three MACE components were evaluated: urgent revascularization, nonfatal MI and complete coronary occlusion by manually reviewing the discharge summary of these patients from the health record. Composite MACE was defined as occurrence of any component. As a clinical reference, we fit a class-balanced logistic-regression baseline from routinely available clinical variables in the test set: age at catheterization, sex, hypertension, diabetes, chronic kidney disease, atrial fibrillation, current or previous smoking, and previous PCI (defined as ≥1 stent documented on angiography). Age was computed as the index procedure date; binary covariates with unknown values were modeled as an explicit missing category. We evaluated three models: the clinical baseline, DeepCORO-CLIP alone (its predicted risk probability) and a combined logistic-regression model integrating the clinical variables above with DeepCORO-CLIP. Each was assessed by patient-level 5-fold grouped cross-validation within the 350-study test cohort, with 1,000-iteration bootstrap confidence intervals.

To assess the ability of DeepCORO-CLIP to estimate LVEF from coronary angiograms^9^, we constructed a paired dataset by linking cardiac catheterization procedures to transthoracic echocardiograms (TTE) using medical record numbers. LVEF, treated as the ground-truth label, was extracted from TTE reports as a continuous value (%). Only catheterization–TTE pairs with an absolute time difference of 30 days or less were retained (median 2 days, interquartile range 1 to 5 days), the same pairing window as in our earlier CathEF study^9^, and we performed sensitivity analysis with tighter windows of ICA–TTE pairs ≤7 days and ≤14 days, respectively. The resulting dataset comprised 7,586 catheterization studies from 6,607 unique patients at the MHI (2017–2023). Data were partitioned at the patient level into training (60%, *n* = 4,382 studies), validation (10%, *n* = 674 studies) and test (30%, *n* = 2,530 studies) sets keeping same patient assignment as in the main experiment. The pretrained DeepCORO-CLIP video encoder served as the backbone. We tested multiple fine-tuning strategies, freezing 75% to 95% of encoder layers and fine-tuning the remaining layers with learning rates ranging from 5 × 10^−6^ to 2 × 10^−5^. For each study, up to four angiographic views were included, with 16 frames per view at 224 × 224 resolution, consistent with our original work. These views were combined using multihead attention pooling with a classification token to enable direct 1:1 comparison. The model was trained jointly for two tasks: continuous LVEF regression using mean absolute error loss, and binary classification of reduced LVEF (LVEF ≤ 40%) using binary cross-entropy loss. Hyperparameters were optimized with Bayesian search and early stopping, using validation loss as the objective. As a baseline, we evaluated CathEF, a previously published model trained on an external UCSF dataset, which predicts LVEF from individual angiographic videos and then averages predictions across views^9^.

### External validation

Our external validation dataset comprised coronary angiograms performed at the UCSF between 2013 and 2019. Coronary stenosis labels were automatically extracted from free-text catheterization reports using DeepSeek-V2.3 (ref. ^22^), a mixture-of-experts LLM deployed via an on-premises containerized Docker pipeline. The model parsed narrative descriptions of coronary anatomy and extracted stenosis percentages for SYNTAX arterial segments, as was done for our internal dataset. Qualitative descriptors were mapped to standardized values using clinical conventions (for example, mild was mapped to ≤30%, moderate to 50%, severe to 70%, critical to 90% and occlusion to 100%). To validate the LLM-derived external labels against cardiologist extraction, we re-extracted a severity-stratified random sample of 236 UCSF reports (4,248 segment labels) with two independent cardiologists and assessed agreement between the cardiologists and the LLM on continuous severity (intraclass correlation coefficient, Pearson correlation, MAE) and the binary obstructive call (≥70%; left main ≥50%; Cohen’s *κ*, sensitivity, specificity).

### Core laboratory adjudication

QCA was performed by two independent interventional cardiologists from MHI blinded to model predictions on 662 randomly selected studies from our test dataset. Each reader assessed percent diameter stenosis for all lesions in the LCA and RCA territories using QCA. Lesions with interreader discrepancies ≥20% were adjudicated by a third reader with 5 years of interventional cardiology experience. The final adjudicated value was used as the reference standard. Interreader agreement was assessed using Cohen’s weighted *κ*. Clinical reports from the original angiographic interpretation served as a secondary comparator representing real-world single-reader performance.

### Prospective single-report reader validation

To evaluate the standalone clinical quality of the free-text reports generated by DeepCORO-CLIP, we performed a prospective, blinded, single-report reader study. From the held-out diagnostic test cohort, we sampled 288 studies with at least one right and two left coronary acquisitions, using ground-truth stratified enrichment so that studies with a stenosis above 50%, a CTO or a thrombus were overrepresented relative to angiographically normal studies (with a minimum of 10% of studies carrying a CTO and 10% carrying a thrombus). Each study was rendered as a standardized grid of up to six diagnostic projections played at each clip’s native frame rate and presented with a single, source-blinded interpretation, either generated automatically by DeepCORO-CLIP or taken from the operator catheterization report, rendered in an identical severity-interval format (below 30%, 30% to 50%, 50% to 70%, 70% to 90%, 90% or greater, occluded; with CTO, thrombus and calcification appended per segment) so that the report source could not be inferred from wording, and uploaded to a web-based annotation platform. Two independent interventional cardiologists with ≥1 year of experience, masked to report source, evaluated each interpretation against the angiographic images and recorded angiogram quality (1 to 4), overall clinical acceptability (ideal, acceptable or unacceptable), three domain scores on a 1 to 4 scale (clinical correctness, completeness and clarity), whether the single most clinically important lesion was correctly identified, and the presence of a clinically important missed finding and of a hallucinated finding, each specified by finding type (stenosis, calcification, CTO, thrombus) and coronary segment. Each of the 288 studies was evaluated at least once, and 77 studies were independently evaluated by both readers to allow estimation of interreader agreement, yielding 365 report evaluations in total: 174 evaluations of DeepCORO-CLIP reports and 191 of operator reports. We also deployed the model using our own bespoke software called PACS-AI^10^ which is detailed in Supplementary Information.

### Statistical analysis

Cross-modal retrieval performance of the contrastive encoder was evaluated in both video-to-text and text-to-video directions, with Recall@K (*K* = 1, 5, 10, 25, 50; *K* is the retrieval rank cutoff, and a correct paired report or video within the top *K* results counts as retrieved) and median rank of the correct report; an alignment score was defined as the mean cosine similarity between each paired video and report embedding. For binary classification tasks, discrimination was assessed using AUROC and AUPRC, with operating-point metrics (sensitivity, specificity, PPV, NPV) at the Youden index. Global and vessel-specific performance were computed as microweighted averages across class-specific metrics. AUROCs were compared using DeLong’s test^23^. For continuous prediction tasks, agreement with reference values was assessed using Pearson correlation *r* and MAE. All 95% CIs were derived from 1,000 bootstrap iterations. Baseline characteristics were compared using two-sample *t*-test (or Wilcoxon rank-sum when normality was not satisfied) for continuous variables and chi-square test (or Fisher’s exact when expected cell counts were small) for categorical variables. To assess multiview integration, we compared three video aggregation strategies: (1) single-video inference, (2) study-level averaging (arithmetic mean across videos) and (3) multivideo attention pooling over 10 randomly sampled videos per study. Performance was evaluated using micro-averaged AUROC across all coronary segments within each territory. End-to-end latency was reported as wall-clock time from study receipt by the PACS-AI listener^10^ to availability of model outputs. All analyses were conducted on the held-out test set and repeated in external validation when available. All models were trained in PyTorch 2.5.1 with CUDA 12.4 on NVIDIA H200 GPUs, using torchvision 0.20.1 and the Transformers library (v4.47.0). For the prospective single-report reader study, categorical outcomes were compared between AI and operator reports using the chi-square test; for 2 × 2 comparisons, the Fisher exact test (odds ratios) and ordinal 1 to 4 scores were compared using the Welch *t*-test and confirmed with the Mann–Whitney *U*-test, and interreader agreement was summarized by percent agreement and Cohen’s *κ* on doubly-read studies, with tests two-sided at an alpha of 0.05 without correction for multiple comparisons. Statistical analyses, including the DeLong test and the bootstrap resampling used for all confidence intervals, were performed in Python 3.11 with scikit-learn 1.7.2, SciPy 1.15.3 and NumPy 2.2.6, and figures were produced with Matplotlib 3.10.8. Model deployment used PACS-AI version 1.0.

### Decision-threshold analysis

To characterize the precision–sensitivity trade-off of the deployed report generator, we swept the per-segment threshold of the study-level binary stenosis head from 0.01 to 0.99 in increments of 0.01 in the internal held-out test set (*n* = 4,828 studies; obstructive disease ≥70% diameter stenosis, ≥50% for the left main), computing sensitivity, specificity, PPV and F1 with 95% CIs from 1,000 bootstrap iterations. We report three operating points: the deployed threshold (0.50), the max-F1 threshold and the balanced (Youden) threshold. The latter two were selected in the same test set in which they are reported, so their metrics are optimistically biased. The released pipeline exposes the per-segment threshold as a user-configurable parameter.

### Reporting summary

Further information on research design is available in the Nature Portfolio Reporting Summary linked to this article.

## Supplementary information

Supplementary Information Supplementary Methods and References.

Reporting Summary

Peer Review file

Supplementary Tables Supplementary Tables 1–15, with one worksheet per table.

## Source data

Source Data Fig. 1 Numerical or image provenance source data corresponding to the panels of this figure.

Source Data Fig. 2 Numerical or image provenance source data corresponding to the panels of this figure.

Source Data Extended Data Fig. 1 Numerical or image provenance source data.

Source Data Extended Data Fig. 2 Numerical or image provenance source data.

Source Data Extended Data Fig. 3 Numerical or image provenance source data.

Source Data Extended Data Fig. 4 Numerical or image provenance source data.

Source Data Extended Data Fig. 5 Numerical or image provenance source data.

Source Data Extended Data Fig. 6 Numerical or image provenance source data.

Source Data Extended Data Fig. 7 Numerical or image provenance source data.

## Data Availability

The datasets analyzed during the current study are not publicly available because of patient privacy concerns but may be available from the corresponding author on reasonable request, as feasible and permitted by the MHI Research Ethics Board. The DeepCORO-CLIP model weights are freely available under the Apache 2.0 license at https://huggingface.co/collections/heartwise/deepcoro-clip. Source data are provided with this paper.

## Extended data

**Extended Data Table 1:** Stenosis quantification by coronary segment

Segment	n ≥70% (%)	MAE (95% CI)	r (95% CI)	AUROC (95% CI)	AUPRC (95% CI)	Sens (95% CI)	Spec (95% CI)	Youden threshold
Left Main	371/4828 (7.7%)	8.4 (8.0–8.9)	0.61 (0.58–0.64)	0.94 (0.93–0.95)	0.64 (0.59–0.69)	0.89 (0.85–0.92)	0.84 (0.83–0.85)	0.0546
Proximal LAD	810/4828 (16.8%)	15.8 (15.3–16.3)	0.64 (0.62–0.67)	0.90 (0.89–0.91)	0.68 (0.64–0.71)	0.86 (0.84–0.88)	0.78 (0.77–0.79)	0.1145
Mid LAD	1040/4828 (21.5%)	18.0 (17.5–18.5)	0.65 (0.62–0.67)	0.89 (0.88–0.90)	0.70 (0.67–0.73)	0.83 (0.81–0.85)	0.77 (0.76–0.79)	0.1754
Distal LAD	160/4828 (3.3%)	4.3 (3.9–4.8)	0.01 (–0.03–0.03)	0.75 (0.72–0.79)	0.11 (0.08–0.14)	0.67 (0.60–0.74)	0.73 (0.71–0.74)	0.0373
D1	537/4828 (11.1%)	14.6 (13.9–15.3)	0.23 (0.20–0.26)	0.76 (0.74–0.77)	0.27 (0.24–0.30)	0.75 (0.72–0.79)	0.63 (0.62–0.65)	0.1308
D2	294/4828 (6.1%)	7.6 (7.0–8.2)	0.12 (0.09–0.15)	0.77 (0.75–0.79)	0.15 (0.13–0.18)	0.82 (0.77–0.86)	0.61 (0.60–0.63)	0.0568
RI	170/4828 (3.5%)	4.7 (4.2–5.1)	0.12 (0.08–0.15)	0.77 (0.74–0.80)	0.10 (0.08–0.13)	0.81 (0.75–0.87)	0.62 (0.61–0.64)	0.0299
Proximal LCX	548/4828 (11.4%)	14.4 (13.9–14.9)	0.56 (0.53–0.59)	0.89 (0.88–0.91)	0.53 (0.48–0.57)	0.89 (0.86–0.92)	0.74 (0.73–0.75)	0.0830
Mid LCX	361/4828 (7.5%)	9.8 (9.2–10.5)	0.06 (0.03–0.09)	0.84 (0.82–0.86)	0.27 (0.23–0.32)	0.86 (0.82–0.89)	0.70 (0.68–0.71)	0.0597
Distal LCX	90/4828 (1.9%)	2.5 (2.2–2.9)	0.12 (0.08–0.15)	0.80 (0.76–0.85)	0.12 (0.07–0.18)	0.90 (0.83–0.95)	0.57 (0.56–0.58)	0.0105
LVP	23/4828 (0.5%)	0.6 (0.4–0.8)	0.00 (–0.03–0.03)	0.93 (0.90–0.96)	0.06 (0.03–0.12)	1.00 (1.00–1.00)	0.74 (0.73–0.75)	0.0723
OM1	307/4828 (6.4%)	8.4 (7.8–9.0)	0.19 (0.16–0.22)	0.79 (0.77–0.81)	0.19 (0.15–0.22)	0.75 (0.69–0.80)	0.71 (0.70–0.73)	0.0767
OM2	340/4828 (7.0%)	8.9 (8.3–9.6)	0.11 (0.08–0.14)	0.81 (0.79–0.83)	0.23 (0.19–0.27)	0.82 (0.78–0.86)	0.67 (0.66–0.68)	0.0014
Proximal RCA	789/4828 (16.3%)	14.2 (13.8–14.7)	0.73 (0.71–0.75)	0.92 (0.91–0.93)	0.73 (0.70–0.76)	0.87 (0.85–0.90)	0.82 (0.81–0.83)	0.1079
Mid RCA	714/4828 (14.8%)	14.1 (13.5–14.6)	0.65 (0.62–0.68)	0.93 (0.92–0.93)	0.69 (0.65–0.72)	0.89 (0.87–0.91)	0.80 (0.79–0.82)	0.1284
Distal RCA	348/4828 (7.2%)	10.3 (9.7–10.9)	0.49 (0.46–0.52)	0.90 (0.88–0.91)	0.46 (0.41–0.52)	0.96 (0.94–0.98)	0.67 (0.66–0.69)	0.0327
PDA	239/4828 (5.0%)	6.9 (6.4–7.5)	0.21 (0.17–0.24)	0.80 (0.77–0.82)	0.18 (0.14–0.23)	0.87 (0.83–0.91)	0.57 (0.56–0.59)	0.0299
Posterolateral	171/4828 (3.5%)	4.7 (4.3–5.2)	0.22 (0.18–0.25)	0.82 (0.79–0.85)	0.14 (0.11–0.18)	0.82 (0.75–0.88)	0.68 (0.67–0.69)	0.0209

Abbreviations: MAE, mean absolute error (%); r, Pearson correlation; AUROC, area under the receiver operating characteristic curve; AUPRC, area under the precision–recall curve; Sens, sensitivity; Spec, specificity; CI, confidence interval; LAD, left anterior descending; LCX, left circumflex; RI: Ramus Intermedius; RCA, right coronary artery; D1/D2, diagonal branches; OM1/OM2, obtuse marginal branches; LVP, left ventricular posterior branch; PDA, posterior descending artery.

**Extended Data Table 2:** Calcification, thrombus and chronic total occlusion classification by coronary segment

Segment	n (%)	AUROC (95% CI)	AUPRC (95% CI)	Sens (95% CI)	Spec (95% CI)	Youden threshold
Left Main	164/4828 (3.4%)	0.88 (0.86–0.91)	0.26 (0.20–0.33)	0.86 (0.80–0.91)	0.77 (0.75–0.78)	0.0230
Proximal LAD	320/4828 (6.6%)	0.81 (0.78–0.83)	0.23 (0.19–0.27)	0.72 (0.67–0.77)	0.76 (0.75–0.77)	0.0729
Mid LAD	271/4828 (5.6%)	0.82 (0.80–0.84)	0.22 (0.18–0.26)	0.72 (0.67–0.77)	0.78 (0.76–0.79)	0.0894
Distal LAD	15/4828 (0.3%)	0.85 (0.76–0.92)	0.04 (0.01–0.09)	0.87 (0.67–1.00)	0.72 (0.71–0.73)	0.0026
D1	66/4828 (1.4%)	0.81 (0.75–0.86)	0.06 (0.04–0.10)	0.73 (0.62–0.84)	0.76 (0.75–0.77)	0.0206
D2	38/4828 (0.8%)	0.88 (0.85–0.92)	0.06 (0.03–0.10)	1.00 (1.00–1.00)	0.63 (0.62–0.65)	0.0065
RI	25/4828 (0.5%)	0.83 (0.77–0.89)	0.02 (0.01–0.04)	0.92 (0.79–1.00)	0.61 (0.59–0.62)	0.0029
Proximal LCX	151/4828 (3.1%)	0.86 (0.84–0.88)	0.19 (0.14–0.25)	0.84 (0.78–0.90)	0.74 (0.72–0.75)	0.0207
Mid LCX	40/4828 (0.8%)	0.84 (0.79–0.89)	0.05 (0.02–0.07)	0.90 (0.79–0.98)	0.69 (0.67–0.70)	0.0062
Distal LCX	10/4828 (0.2%)	0.90 (0.80–0.98)	0.05 (0.01–0.14)	0.81 (0.50–1.00)	0.88 (0.87–0.89)	0.0050
LVP	4/4828 (0.1%)	0.92 (0.82–0.98)	0.01 (0.00–0.03)	1.00 (1.00–1.00)	0.81 (0.80–0.83)	0.0104
OM1	28/4828 (0.6%)	0.87 (0.83–0.91)	0.09 (0.02–0.21)	0.86 (0.71–0.97)	0.78 (0.77–0.80)	0.0091
OM2	33/4828 (0.7%)	0.86 (0.80–0.91)	0.05 (0.03–0.10)	0.88 (0.75–0.97)	0.70 (0.69–0.72)	–
Proximal RCA	279/4828 (5.8%)	0.85 (0.83–0.87)	0.25 (0.21–0.30)	0.87 (0.83–0.91)	0.70 (0.69–0.72)	0.0376
Mid RCA	169/4828 (3.5%)	0.87 (0.85–0.89)	0.20 (0.15–0.25)	0.92 (0.88–0.96)	0.71 (0.70–0.72)	0.0302
Distal RCA	71/4828 (1.5%)	0.90 (0.87–0.92)	0.11 (0.07–0.14)	0.87 (0.79–0.94)	0.81 (0.80–0.82)	0.0171
PDA	23/4828 (0.5%)	0.87 (0.82–0.92)	0.08 (0.02–0.17)	0.83 (0.66–0.96)	0.80 (0.79–0.81)	0.0066
Posterolateral	17/4828 (0.4%)	0.85 (0.79–0.92)	0.02 (0.01–0.05)	1.00 (1.00–1.00)	0.62 (0.60–0.63)	0.0012

a, Calcification; n denotes segments with moderate or severe calcification divided by the total.

b, Thrombus; n denotes thrombus-positive segments divided by the total. LVP is excluded because no thrombus cases were present.

c, Chronic total occlusion; n denotes positive segments divided by the total. Values in parentheses are 95% confidence intervals. AUROC, area under the receiver operating characteristic curve; AUPRC, area under the precision-recall curve; Sens, sensitivity; Spec, specificity; LAD, left anterior descending; LCX, left circumflex; RCA, right coronary artery; RI, ramus intermedius; D1/D2, diagonal branches; OM1/OM2, obtuse marginal branches; LVP, left ventricular posterior branch; PDA, posterior descending artery; CTO, chronic total occlusion.

## Code availability

The underlying code for DeepCORO-CLIP for training and inference is available for noncommercial research use under the MHI research use agreement in a GitHub repository at https://github.com/HeartWise-AI/DeepCORO_CLIP.

## References

[1] Virani, S. S. et al. 2023 AHA/ACC/ACCP/ASPC/NLA/PCNA guideline for the management of patients with chronic coronary disease: a report of the American Heart Association/American College of Cardiology Joint Committee on Clinical Practice Guidelines. *Circulation* **148**, e9–e119 (2023). 10.1161/CIR.0000000000001168 37471501

[2] Shah, R. et al. Comparison of visual assessment of coronary stenosis with independent quantitative coronary angiography: findings from the Prospective Multicenter Imaging Study for Evaluation of Chest Pain (PROMISE) trial. *Am. Heart J.* **184**, 1–9 (2017). 10.1016/j.ahj.2016.10.014 PMC5785086 27892881

[3] Leape, L. L. et al. Effect of variability in the interpretation of coronary angiograms on the appropriateness of use of coronary revascularization procedures. *Am. Heart J.* **139**, 106–113 (2000). 10.1016/s0002-8703(00)90316-8 10618570

[4] Nallamothu, B. K. et al. Comparison of clinical interpretation with visual assessment and quantitative coronary angiography in patients undergoing percutaneous coronary intervention in contemporary practice. *Circulation* **127**, 1793–1800 (2013). 10.1161/CIRCULATIONAHA.113.001952 PMC3908681 23470859

[5] Avram, R. et al. CathAI: fully automated coronary angiography interpretation and stenosis estimation. *npj Digit. Med.* **6**, 142 (2023). 10.1038/s41746-023-00880-1 PMC10421915 37568050

[6] Langlais, É. L. et al. Evaluation of stenoses using AI video models applied to coronary angiography. *npj Digit. Med.* **7**, 138 (2024). 10.1038/s41746-024-01134-4 PMC11116436 38783037

[7] Vukadinovic, M. et al. Comprehensive echocardiogram evaluation with view primed vision language AI. *Nature* **650**, 970–977 10.1038/s41586-025-09850-x (2026). PMC12935550 41219498

[8] Christensen, M., Vukadinovic, M., Yuan, N. & Ouyang, D. Vision–language foundation model for echocardiogram interpretation. *Nat. Med.* **30**, 1481–1488 10.1038/s41591-024-02959-y (2024). PMC11108770 38689062

[9] Avram, R. et al. Automated assessment of cardiac systolic function from coronary angiograms with video-based artificial intelligence algorithms. *JAMA Cardiol.* **8**, 586–594 (2023). 10.1001/jamacardio.2023.0968 PMC10267763 37163297

[10] Theriault-Lauzier, P. et al. A responsible framework for applying artificial intelligence on medical images and signals at the point-of-care: the PACS-AI platform. *Can. J. Cardiol.* **40**, 1828–1840 10.1016/j.cjca.2024.05.025 (2024). 38885787

[11] Hideo-Kajita, A. et al. Impact of two formulas to calculate percentage diameter stenosis of coronary lesions: from stenosis models (phantom lesion model) to actual clinical lesions. *Int. J. Cardiovasc. Imaging* **35**, 2139–2146 (2019). 10.1007/s10554-019-01672-z 31352559

[12] Du, T. et al. Training and validation of a deep learning architecture for the automatic analysis of coronary angiography. *Eurointervention* **17**, 32–40 (2021). 10.4244/EIJ-D-20-00570 PMC9753915 32830647

[13] Munim, A., et al. EchoJEPA: a latent predictive foundation model for echocardiography. Preprint at https://arxiv.org/abs/2602.02603 (2026).

[14] Ndrepepa, G. et al. Association of progression or regression of coronary artery atherosclerosis with long-term prognosis. *Am. Hear. J.* **177**, 9–16 (2016). 10.1016/j.ahj.2016.03.016 27297844

[15] D’Ascenzo, F. et al. Machine learning-based prediction of adverse events following an acute coronary syndrome (PRAISE): a modelling study of pooled datasets. *Lancet* **397**, 199–207 (2021). 10.1016/S0140-6736(20)32519-8 33453782

[16] Batra, G. et al. Biomarker-based prediction of recurrent ischemic events in patients with acute coronary syndromes. *J. Am. Coll. Cardiol.* **80**, 1735–1747 (2022). 10.1016/j.jacc.2022.08.767 36302586

[17] Kumar, R. et al. Assessment of the prognostic performance of TIMI, PAMI, CADILLAC and GRACE scores for short-term major adverse cardiovascular events in patients undergoing emergent percutaneous revascularisation: a prospective observational study. *BMJ Open* **15**, e091028 (2025). 10.1136/bmjopen-2024-091028 PMC11904351 40074268

[18] Bogner, E. et al. Comprehensive machine learning-enabled outcome prediction for patients with coronary artery disease using multicentre patient data. *Can. J. Cardiol.* **42**, 1384–1396 10.1016/j.cjca.2025.12.024 (2026). 41421637

[19] Collins, G. S. et al. TRIPOD+AI statement: updated guidance for reporting clinical prediction models that use regression or machine learning methods. *Brit. Med. J.* **385**, e078378 (2024). 10.1136/bmj-2023-078378 PMC11019967 38626948

[20] Mario, C. D. & Sutaria, N. Coronary angiography in the angioplasty era: projections with a meaning. *Heart* **91**, 968 (2005). 10.1136/hrt.2005.063107 PMC1768997 15958378

[21] Georgios, S. et al. The SYNTAX Score: an angiographic tool grading the complexity of coronary artery disease. *EuroIntervention* **1**, 219–227 (2005). 19758907

[22] DeepSeek-AI, et al. DeepSeek-V2: a strong, economical, and efficient mixture-of-experts language model. Preprint at https://arxiv.org/abs/2405.04434 (2024).

[23] DeLong, E. R., DeLong, D. M. & Clarke-Pearson, D. L. Comparing the areas under two or more correlated receiver operating characteristic curves: a nonparametric approach. *Biometrics* **44**, 837–845 (1988). 3203132

