# Supplementary material for: A multiview video–text model detects obstructive coronary stenosis and predicts 1-year cardiovascular events from angiography
Source: Nat Cardiovasc Res. 2026 Oct 13;5(11):1179–92. doi: 10.1038/s44161-026-00890-8 (PMC13759332; doi:10.1038/s44161-026-00890-8)
Supplement: Supplementary file 1 [file 44161_2026_890_MOESM1_ESM.pdf]

nature cardiovascular research

Supplementary information

https://doi.org/10.1038/s44161-026-00890-8

A multiview video–text model detects obstructive coronary stenosis and predicts 1-year cardiovascular events from angiography

In the format provided by the authors and unedited

Supplementary Methods

Phase 1: Contrastive Learning for Pretraining the video encoder

DeepCORO-CLIP employed a dual-encoder contrastive learning architecture to align single diagnostic invasive coronary angiography (ICA) videos with the side-specific text of their parent procedural report in a shared 512-dimensional embedding space, with each video paired only to the LCA or RCA portion of the report describing the coronary system it imaged. This report contains descriptions of the stenoses, presence or absence of calcification, thrombus or CTOs by artery segment as well as coronary dominance. This local pairing avoided the spurious gradient that whole-report concatenation would introduce by forcing right-system videos to align with left-system findings. The video encoder used a Multi-scale Vision Transformer v2 (MViTv2-S) backbone (TorchVision implementation) pretrained on Kinetics-400.[1],[2] Videos were sampled at 7.5 to 15 fps and resized to 224×224 pixels. The backbone comprised 16 transformer blocks distributed across four stages, using pooling attention for hierarchical spatiotemporal modeling. [1] The text encoder utilized BioMedBert-base (12 layers, 768 dimensions), with weights pretrained from PubMed, with mean pooling of token embeddings.[2] We performed a Bayesian hyperparameter[3] optimization sweep across model architectures (MViT, X3D), temporal sampling strategies (16 to 64 frames, stride 1 to 3), loss functions (Contrastive[4] , SigLip[5] and InfoNCE[6]), learning rates (5.8×10^{-6} to 8.0×10^{-5}), encoder freeze ratios (video 72.8 to 94.8%, text 60.7 to 90.2%), and batch configurations (12 to 64) to identify optimal pretraining configurations for downstream clinical tasks (Extended Data Fig. 8). For each study, diagnostic videos were split into left and right coronary videos. Video-report pairs were constructed such that left coronary videos were aligned with left coronary territory segments (adjusted for coronary dominance) and right coronary videos with corresponding right coronary segments from the same report (Figure 1b). The final model architecture and hyperparameters were selected based on a combination of lowest validation loss and highest recall and alignment score between the video and text features. (Supplementary Table 9). To test our encoder's zero-shot capabilities, we performed a prompt-free retrieval probe by freezing the contrastive encoder and, on the held-out set, ranked all 4,851 candidate report embeddings against each video embedding (and the reverse) by cosine similarity in the shared latent space, computing Recall@K and median rank

without any task-specific fine-tuning or text prompting; same-patient discrimination was assessed by comparing study-level mean cosine similarity for same-patient versus different-patient angiogram pairs.

Phase 2: Task-specific fine-tuning

Following pretraining, we evaluated DeepCORO-CLIP on downstream clinical tasks using linear probing with task-specific classification heads (Supplementary Table 10). The pretrained video encoder extracted embeddings (Phase 1) from up to 10 videos per study, which were aggregated via an attention-based multi-video pooling module with a learnable classification (CLS) token and passed to task-specific MLP prediction heads. This enabled the model to "visualise" all of the diagnostic coronary angiogram videos. The pretrained video encoder was evaluated either with all weights frozen or with up to the deepest 20% of layers unfrozen, using a learning rate tenfold lower than the main learning rate. Across configurations, the multi-instance attention pooling module and task-specific prediction heads were trained using a learning rate of 5.5×10^{-6}. For multi-video inputs, up to 10 videos per study (zero padded if less than 10) were processed through the frozen video encoder, generating embeddings that were aggregated using attention-based pooling[7] with a learnable classification (CLS) token representing a study-level aggregate of the videos' features.[8] We explored multi-video fusion strategies using cross-attention with CLS tokens [8], gated attention pooling, mean pooling, max pooling, and hybrid approaches that concatenate multiple aggregation methods (i.e. attention + CLS and mean + CLS). For hierarchical processing of patch-level features, we employed two-stage CLS aggregation where within-video CLS tokens first aggregate patch embeddings before a study-level CLS token aggregates across video representations, with separate learning rates controlling within-video (4.1×10^{-3}) and across-video (2.0×10^{-3}) attention components. For hybrid methods, multiple instance learning framework produced importance weights for each video in relation to predictions of specific coronary segments and pathologies, using weighted averages of video embeddings for final predictions with CLS token aggregation. [9,10] Video embeddings were pooled across temporal dimensions before cross-video attention, with separate within-video and across-video attention modules (learning rates 4.1×10^{-3} and 2.0×10^{-3} respectively, weight decay 6.3×10^{-5} and 6.8×10^{-6}). Task-specific heads were single-layer linear classifiers (dropout $p = 0.2$) trained for 50 epochs using AdamW[11] optimizer with warm restarts (cycles). Binary

classification tasks (CTO, thrombus, calcification, stenosis ≥70%) used binary cross entropy loss with logits (learning rate 3×10^{-4}, weight decay 1×10^{-5}). Stenosis regression tasks used Huber loss[12] (learning rate 5×10^{-4}, weight decay 5×10^{-6} with loss weights twice as high as the other heads). Videos were randomly shuffled during training and sampled at 16 frames with random augmentations (rotation, distortion, random patch masking) using RandAugment package.[13] All experiments used mixed precision training (FP16) with seed 42 for reproducibility. Task-specific fine-tuning maintained the same patient-level dataset split (70/15/15% training/validation/test) as the contrastive learning phase, sampling up to 10 videos per study when available. In the final analyses, we report results for three configurations: (1) a fully frozen encoder, (2) a model with 80% of encoder layers frozen with 20% of the remaining final layers unfrozen and fine-tuned (the best performing configuration), and (3) a variant incorporating a 12 × 1 view embedding matrix (Supplementary Table 1) as an additional feature vector input to encode angiographic projection information. For additional comparison, we evaluated a domain-adapted EchoJEPA model[14] , which employs V-JEPA 2's latent predictive pretraining objective with a ViT-Large encoder (303M parameters) pretrained on the same coronary angiography training corpus for 200 epochs followed by 60 epochs of learning rate annealing, using up to 10 videos per study with the same multi-view early fusion and attentive probing framework as our multi-instance learning approach as above. We report the performance of an EchoJEPA frozen encoder with lightweight attentive probes (EJ-Frozen) trained for 20 epochs on the same multi-task stenosis regression and binary classification targets using identical patient-level splits and labels as the core DeepCORO-CLIP model.

Encoder Probing and Visualisation

We extracted 512-dimensional embeddings from all coronary angiography videos in the held-out test set using the trained MViT-v2 encoder (34.5M encoder), with each video uniformly sampled to 16 frames and resized to 224×224 pixels. To visualize the learned embedding space, we applied t-distributed Stochastic Neighbor Embedding (t-SNE) [15] with perplexity 30 following principal component analysis dimensionality reduction to 50 components for computational efficiency.[16] We performed clustering analysis using k-means on the raw 512-dimensional embeddings to identify distinct patterns in the embedding space, evaluating cluster quality using silhouette scores and the elbow method. To characterize the clinical and anatomical organization

of the learned representations, we conducted sub-analyses stratified by stenosis severity (quantified as peak percent diameter stenosis across all coronary segments per study), coronary artery present (left versus right coronary system), and cardiac dominance pattern (right dominant, left dominant, or co-dominant). Stenosis severity was extracted from angiogram reports using regular expression pattern matching, categorizing studies into normal (0%), mild (1 to 49%), moderate (50 to 69%), severe (70 to 99%), and chronic total occlusion (100%). Moreover, to see if the embeddings can be used to track progression of disease, study-level embeddings were extracted from the test set using the DeepCORO-CLIP encoder. Among the cohort, 352 patients (9.2%) had longitudinal follow-up with multiple diagnostic catheterizations in the test set; only consecutive study pairs were included for this analysis. Disease status between consecutive studies was classified as: stable (no vessel with ≥20% absolute stenosis change), progressed (new stenosis >50% or ≥20% worsening), or improved (≥20% stenosis decreases without concurrent progression). Individual video sequences were encoded into 512-dimensional vectors via MViT-v2 backbone and aggregated into 1024-dimensional study-level embeddings through the attention/CLS token pooling. Embeddings were extracted from the penultimate layer prior to final projection. Embedding distance was defined as (1 - cosine similarity), where 0 indicates identical representations [range 0,1]. For patients undergoing PCI, post-procedural embeddings were used to reflect coronary anatomy after intervention.

Supervised MViT Baseline Comparator

To benchmark DeepCORO-CLIP pre-trained regimen against a strong supervised comparator, we trained an MViT-v2 video model initialized from Kinetics and fine-tuned end-to-end for stenosis interpretation with 100% of layers unfrozen. The model used the same patient-level train/validation/test split (70/15/15), identical preprocessing and augmentations, and the same multi-video study sampling policy as DeepCORO-CLIP. This baseline isolates the incremental value of video-text contrastive pretraining and cross-modal alignment beyond fully supervised fine-tuning.

Deployment

We describe the deployment of DeepCORO-CLIP within a PACS-integrated inference system (PACS-AI)[17,18] running on-premises inside the hospital network. Coronary angiography studies

flowed as DICOM directly from the catheterization laboratory to the clinical PACS, while a parallel DICOM listener automatically received incoming studies and triggered near real-time inference once diagnostic series were available. PACS-AI performed automated preprocessing (DICOM decoding, frame sampling, resizing, and diagnostic-phase selection), embedded up to a fixed number of diagnostic videos per study using the DeepCORO-CLIP encoder, and generated study-level outputs via attention-based multi-video pooling. Inference was performed on a single NVIDIA RTX A6000 GPU, and the deployment was containerized with versioned logging of preprocessing decisions and latency to support traceability and operational monitoring.

Supplementary References

1. Li, Y. *et al.* MViTv2: Improved Multiscale Vision Transformers for Classification and Detection. *arXiv* https://doi.org/10.48550/arxiv.2112.01526 (2021) doi:10.48550/arxiv.2112.01526.

2. Gu, Y. *et al.* Domain-Specific Language Model Pretraining for Biomedical Natural Language Processing. *ACM Trans. Comput. Healthc. (Heal.)* **3**, 1–23 (2021).

3. Onorato, G. Bayesian Optimization for Hyperparameters Tuning in Neural Networks. *arXiv* https://doi.org/10.48550/arxiv.2410.21886 (2024) doi:10.48550/arxiv.2410.21886.

4. Radford, A. *et al.* Learning Transferable Visual Models From Natural Language Supervision. *arXiv* https://doi.org/10.48550/arxiv.2103.00020 (2021) doi:10.48550/arxiv.2103.00020.

5. Zhai, X., Mustafa, B., Kolesnikov, A. & Beyer, L. Sigmoid Loss for Language Image Pre-Training. *arXiv* https://doi.org/10.48550/arxiv.2303.15343 (2023) doi:10.48550/arxiv.2303.15343.

6. Oord, A. van den, Li, Y. & Vinyals, O. Representation Learning with Contrastive Predictive Coding. *arXiv* https://doi.org/10.48550/arxiv.1807.03748 (2018) doi:10.48550/arxiv.1807.03748.

7. Vaswani, A. *et al.* Attention Is All You Need. *Arxiv* (2017).

8. Wang, A. *et al.* [CLS] Token Tells Everything Needed for Training-free Efficient MLLMs. *arXiv* https://doi.org/10.48550/arxiv.2412.05819 (2024) doi:10.48550/arxiv.2412.05819.

9. Quellec, G., Cazuguel, G., Cochener, B. & Lamard, M. Multiple-Instance Learning for Medical Image and Video Analysis. *IEEE Rev. Biomed. Eng.* **10**, 213–234 (2017).

10. Ilse, M., Tomczak, J. M. & Welling, M. Attention-based Deep Multiple Instance Learning. *arXiv* https://doi.org/10.48550/arxiv.1802.04712 (2018) doi:10.48550/arxiv.1802.04712.

11. Loshchilov, I. & Hutter, F. Decoupled Weight Decay Regularization. *arXiv* https://doi.org/10.48550/arxiv.1711.05101 (2017) doi:10.48550/arxiv.1711.05101.

12. Meyer, G. P. An Alternative Probabilistic Interpretation of the Huber Loss. *arXiv* https://doi.org/10.48550/arxiv.1911.02088 (2019) doi:10.48550/arxiv.1911.02088.

13. Cubuk, E. D., Zoph, B., Shlens, J. & Le, Q. V. RandAugment: Practical automated data augmentation with a reduced search space. *Arxiv* https://doi.org/10.48550/arxiv.1909.13719 (2019) doi:10.48550/arxiv.1909.13719.

14. Munim, A. *et al.* EchoJEPA: A Latent Predictive Foundation Model for Echocardiography. *arXiv* https://doi.org/10.48550/arxiv.2602.02603 (2026) doi:10.48550/arxiv.2602.02603.

15. Cai, T. T. & Ma, R. Theoretical Foundations of t-SNE for Visualizing High-Dimensional Clustered Data. *arXiv* https://doi.org/10.48550/arxiv.2105.07536 (2021) doi:10.48550/arxiv.2105.07536.

16. Shlens, J. A Tutorial on Principal Component Analysis. *arXiv* https://doi.org/10.48550/arxiv.1404.1100 (2014) doi:10.48550/arxiv.1404.1100.

17. Avram, R. *et al.* PACS-AI : Artificial Intelligence Operating System for Medical Imaging. www.pacsai.co (2025).

18. Theriault-Lauzier, P. *et al.* A responsible framework for applying artificial intelligence on medical images and signals at the point-of-care: the PACS-AI platform. *Can. J. Cardiol.* https://doi.org/10.1016/j.cjca.2024.05.025 (2024) doi:10.1016/j.cjca.2024.05.025.
