# Supplementary material for: A multiview video–text model detects obstructive coronary stenosis and predicts 1-year cardiovascular events from angiography
Source: Nat Cardiovasc Res. 2026 Oct 13;5(11):1179–92. doi: 10.1038/s44161-026-00890-8 (PMC13759332; doi:10.1038/s44161-026-00890-8)
Supplement: Supplementary file 3 [file 44161_2026_890_MOESM3_ESM.pdf]

nature portfolio

Peer Review File

A multi-view video-text foundation model detects obstructive coronary stenosis and predicts one-year cardiovascular events from angiography

Corresponding Author: Dr Robert Avram

This file contains all reviewer reports in order by version, followed by all author rebuttals in order by version.

Version 1:

Reviewer comments:

Reviewer #1

(Remarks to the Author)
I have no further comments

(Remarks on code availability)

Reviewer #2

(Remarks to the Author)
Thank you for your revision, I have no further comments at this point.

(Remarks on code availability)

Reviewer #3

(Remarks to the Author)
Comment 1:
The abstract contains too few supporting numbers. The statements on one-year cardiovascular-event prediction, ejection-fraction prediction, and automated report generation should each be backed by the corresponding figures from the main text.
Comment 2:
A quarter of AI-generated reports being rated unacceptable is a substantial fraction, particularly given that these cases were concentrated in missed proximal obstructive stenoses. The authors note that recalibration would improve sensitivity for these findings. Additional analysis identifying the optimal precision–sensitivity trade-off, or code allowing users to adjust this balance themselves, would strengthen the paper.
Comment 3 (minor):
The conclusion contains an incomplete sentence:
"With publicly released code, weights, and deployment infrastructure, and a prospective reader study showing that its reports are clinically usable in most studies and err..."
This sentence is cut off and should be completed.

(Remarks on code availability)
Same as before

Version 2:

Reviewer comments:

Reviewer #3

(Remarks to the Author)
No further comments

(Remarks on code availability)

Open Access This Peer Review File is licensed under a Creative Commons Attribution 4.0 International License, which permits use, sharing, adaptation, distribution and reproduction in any medium or format, as long as you give appropriate credit to the original author(s) and the source, provide a link to the Creative Commons license, and indicate if changes were made.
In cases where reviewers are anonymous, credit should be given to 'Anonymous Referee' and the source.
The images or other third party material in this Peer Review File are included in the article's Creative Commons license, unless indicated otherwise in a credit line to the material. If material is not included in the article's Creative Commons license and your intended use is not permitted by statutory regulation or exceeds the permitted use, you will need to obtain permission directly from the copyright holder.
To view a copy of this license, visit https://creativecommons.org/licenses/by/4.0/

Response to Editors and Reviewers

We thank the editor and the three reviewers for their continued engagement. Reviewers #1 and #2 have no further comments, and Reviewer #3's three remaining points are addressed below. All changes are highlighted in the revised manuscript and a marked-up copy is provided separately. Two new display items answer Comment 2: a per-segment decision-threshold sweep (Extended Data Fig. 3) and the corresponding operating-point table with 95% confidence intervals (Extended Data Table 7). The reader study is now reported as Table 5 in the main manuscript, and the remaining display items are split between Extended Data and Supplementary Information. Reviewer comments are reproduced in italics and our responses follow each comment.

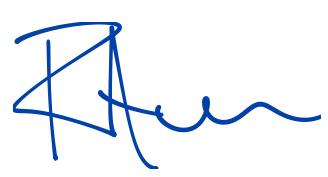

Robert Avram, M.D. F.R.C.P. (C) D.R.C.P.S. (C), M.Sc.
Interventional Cardiologist
Heartwise (heartwise.ai), Montreal Heart Institute
FRQS Junior 2 Scholar
Clinical associate professor, University of Montreal
Associate Editor in Artificial Intelligence, Circulation

Response to editors' comments

1. Manuscript format. The revised manuscript is supplied as a Word file with all changes shown as tracked changes.

2. Title. The colon and parentheses have been removed. The revised title is "A multi-view video-text foundation model detects obstructive coronary stenosis and predicts one-year cardiovascular events from angiography", 139 characters including spaces, with no punctuation, stating the principal findings.

3. Abstract accessibility and length. The abstract has been rewritten to 160 words. It opens with the clinical background in non-technical terms, retains the "Here we show" construction, and carries supporting figures for every claim (see Reviewer #3, Comment 1). Invasive coronary angiography is glossed as an X-ray study of the heart arteries, ejection fraction as cardiac pump function, and AUROC is spelled out before the abbreviation is used.

4. Data archiving and Data Availability statement. A standalone Data Availability section now appears after the Methods and before the References. Because the source data are identifiable clinical imaging studies, they cannot be deposited in a public repository; the statement specifies what is available, from whom, and under what institutional restrictions. Model weights will be released at the stated Hugging Face collection upon publication.

5. Code availability. A standalone Code Availability section likewise precedes the References. It documents that the released report-generation pipeline exposes the per-segment decision threshold as a user-configurable parameter, supplies the operating points of Extended Data Table 7 as selectable presets, and includes the threshold-sweep code (see Reviewer #3, Comment 2).

6a. Main display items. The prospective reader-study results support an abstract-level conclusion but had no display item; they are now Table 5. To stay within the eight-item main limit, the two subgroup tables were merged into a two-panel Table 3 (panel a, lesion detection; panel b, stenosis regression) and the MACE table renumbered Table 4. The main paper now comprises two figures and five tables, and no result has been removed.

6b. Extended Data. The previous version cited 22 Extended Tables and 7 Extended Figures. These have been triaged to exactly 10 Extended Data items, with the remainder moved to Supplementary Information and all in-text citations renumbered. The full mapping is in Appendix A.

7. Statistics and error bars. All confidence intervals are defined in the figure and table legends as 95% bootstrap confidence intervals with the number of iterations stated (1,000), and the sample size is given in each legend. Discrimination metrics are reported to two decimal places and labelled AUROC.

8. Figure legends. The legends for the two new Extended Data items define every symbol and error bar and state the sample size and the definition of obstructive disease. All legends are under 350 words and abbreviations are defined.

9. Competing interests. Retained and unchanged.

10. Author contributions and correspondence. The contribution statement is retained, and a statement naming the author to whom correspondence and requests for materials should be addressed has been added ahead of the back matter. The submitted version had no Acknowledgements; an Acknowledgements and funding statement follows the correspondence statement.

11. Reporting Summary. A completed Reporting Summary form is uploaded with this revision.

12. References. The reference list has been checked against the 50-reference guideline for Articles, excluding citations appearing only in the Methods.

Response to Reviewer #3

Comment 1
The abstract contains too few supporting numbers. The statements on one-year cardiovascular-event prediction, ejection-fraction prediction, and automated report generation should each be backed by the corresponding figures from the main text.

Response. We agree and have added the corresponding figures to all three statements. The abstract now reports one-year cardiovascular events as AUROC 0.79 (95% CI 0.74 to 0.84) versus 0.65 for clinical variables alone, matching Table 4 and Figure 2C; ejection fraction, described as cardiac pump function so the sentence is intelligible outside cardiology, as a mean absolute error of 7.3% versus 8.8% for a previous model, matching Extended Data Table 4; and automated report generation as clinically usable in 76% of evaluations versus 88% for cardiologists, without more fabricated findings (17% versus 14%, p=0.38), matching the reader-study Results. The stenosis statement retains AUROC 0.89 internally and externally, and AUROC is now spelled out at first use. The revised abstract is 160 words, as close to the 150-word guidance as the added figures allow; we would be glad to trim further if the editor prefers.

Comment 2
A quarter of AI-generated reports being rated unacceptable is a substantial fraction, particularly given that these cases were concentrated in missed proximal obstructive stenoses. The authors note that recalibration would improve sensitivity for these findings. Additional analysis identifying the optimal precision–sensitivity trade-off, or code allowing users to adjust this balance themselves, would strengthen the paper.

Response. We have done both of the things suggested: we added the quantitative trade-off analysis, and we made the balance user-adjustable in the released code.

(a) New analysis. We swept the decision threshold of the study-level binary stenosis head from 0.01 to 0.99 in increments of 0.01, independently for each coronary segment, in the internal held-out test set (n = 4,828 studies; obstructive disease defined as ≥70% diameter stenosis and ≥50% for the left main), computing sensitivity, specificity, positive predictive value and F1 with 95% bootstrap confidence intervals. We report three operating points per segment: the deployed threshold (0.50), the maximum-F1 threshold, and the Youden-balanced threshold. These appear as Extended Data Fig. 3 and Extended Data Table 7, with new Methods and Results subsections.

Across the six segments that accounted for the reader-study misses (left main and the proximal and mid segments of the major vessels; prevalence 7.7% to 21.5%):

Operating point	Threshold	Sensitivity	Specificity	PPV
**Deployed**	0.50	0.39 to 0.62	0.94 to 0.98	0.58 to 0.74
**Maximum F1**	0.23 to 0.39	0.60 to 0.77	0.83 to 0.97	0.44 to 0.70
**Balanced (Youden)**	0.06 to 0.18	0.83 to 0.90	0.74 to 0.85	0.30 to 0.51

The proximal misses observed in the reader study therefore reflect the deployed operating threshold rather than absent model signal: the same model detects 39% to 62% of obstructive proximal lesions at the deployed threshold and 83% to 90% at a balanced threshold, at the cost of precision. The trade-off is steepest where prevalence is lowest; in the left main (prevalence 7.7%), moving from the deployed to the balanced threshold raises sensitivity from 0.51 (95% CI 0.47 to 0.56) to 0.87 (0.83 to 0.90) but reduces PPV from 0.68 (0.63 to 0.74) to 0.32 (0.29 to 0.35), meaning roughly two of every three alarms would be false. We therefore present the maximum-F1 point as an intermediate compromise rather than recommending a single optimal threshold. Both the maximum-F1 and Youden thresholds were selected in the same held-out set in which they are evaluated, so those metrics are optimistically biased; this caveat is stated in the Methods, Results, Discussion and Limitations, and the analysis is framed as characterising the achievable trade-off rather than validating a specific operating point.

(b) User-adjustable code. The released report-generation pipeline (https://github.com/HeartWise-AI/DeepCORO_CLIP) now exposes the per-segment decision threshold as a user-configurable parameter: the deployed value (0.50) remains the default, the maximum-F1 and balanced thresholds of Extended Data Table 7 are provided as presets, and the threshold-sweep code is included so that a site can re-derive its own operating points. This is documented in the new Code Availability section and referenced in the Discussion.

(c) Consequent correction. The Discussion sentence stating that per-vessel sensitivity for significant proximal lesions "rose from 0.48 to 0.84 at a balanced threshold" has been replaced by the segment-resolved figures above, which are traceable to Extended Data Fig. 3 and Extended Data Table 7.

Comment 3 (minor)
The conclusion contains an incomplete sentence: "With publicly released code, weights, and deployment infrastructure, and a prospective reader study showing that its reports are clinically usable in most studies and err..." This sentence is cut off and should be completed.

Response. The sentence was truncated in the version the reviewer received. It is complete in the revised manuscript and now carries the supporting figure rather than the vaguer "most studies":

With publicly released code, weights, and deployment infrastructure, and a prospective reader study showing that its reports are clinically usable in 76% of evaluations and err through conservative omission rather than fabrication, DeepCORO-CLIP provides a foundation for human-in-the-loop angiographic interpretation and a building block for multimodal cardiovascular AI systems.

Reviewer #3, remarks on code availability
The code-availability remark in the decision letter we received was blank. The repository at https://github.com/HeartWise-AI/DeepCORO_CLIP contains the training, inference and report-generation code; the report-generation pipeline now exposes the per-segment decision threshold as a user-configurable parameter with documented presets; and the threshold-sweep analysis reported in Extended Data Fig. 3 and Extended Data Table 7 is included so that the results can be reproduced and re-derived locally. We would be glad to address any specific point that did not reach us. We also have the CodeOcean working properly, generating predictions on 10 exams.

Additional corrections made during this revision
We identified and corrected several internal inconsistencies while preparing this revision. None affects the study's conclusions.

1. The Discussion quoted binary stenosis discrimination as AUROC 0.87 where the Results report micro-AUROC 0.89. Corrected to 0.89.

2. The reader-study Methods described half (n=250) AI and half (n=250) operator reports among 288 studies, which is arithmetically inconsistent. The Methods now match the Results: 288 unique angiograms and 365 evaluations, comprising 174 AI and 191 operator reports, with 77 studies doubly read.

3. A placeholder in the LVEF Methods has been replaced with the intended citation.

4. A Pearson correlation reported as 0.650 is now reported as 0.65, consistent with the rest of the manuscript.

5. Discrimination metrics previously expressed as percentages are now two-decimal proportions labelled AUROC throughout.

Appendix A. Extended Data renumbering map

The manuscript previously cited 22 Extended Tables and 7 Extended Figures. The revised version cites exactly 10 Extended Data items, listed below; all remaining items are relabelled Supplementary. The two new items arising from Reviewer #3's Comment 2 occupy Extended Data Fig. 3 and Extended Data Table 7.

Extended Data item	Source
**Fig. 1**	old Extended Figure 1 (cohort flow)
**Fig. 2**	old Extended Figure 7 (reader-study design)
**Fig. 3**	new (threshold sweep, Comment 2)
**Table 1**	old Extended Table 9 (segment-level stenosis metrics)
**Table 2**	old Extended Tables 10, 11, 12 consolidated (calcification, thrombus, CTO)
**Table 3**	old Extended Tables 14, 15 consolidated (Kinetics-400 MViT baseline)
**Table 4**	old Extended Table 18 (LVEF versus CathEF)
**Table 5**	old Extended Tables 19, 20 consolidated (core-laboratory adjudication)
**Table 6**	old Extended Table 22 (external validation, UCSF)
**Table 7**	new (operating points, Comment 2)

Reassigned to Supplementary Information: old Extended Tables 1 to 5 become Supplementary Tables 1 to 5; old Extended Table 8 becomes Supplementary Table 6; old Extended Table 14 (vendor subgroup) becomes Supplementary Table 7; old Extended Table 21 becomes Supplementary Table 8; and old Extended Figures 3, 4, 5 and 6 become Supplementary Figs. 1 to 4. All in-text citations have been renumbered and no legacy labels remain.

Old Extended Tables 6, 7, 13, 16 and 17 and old Extended Figure 2 are cited nowhere in the main text of the submitted version. We placed them in Supplementary Information.
